# Compounds Reducing Human Sperm Motility as Potential Nonhormonal Contraceptives Identified Using a High-Throughput Phenotypic Screening Platform

**DOI:** 10.1021/acsomega.5c08336

**Published:** 2025-11-27

**Authors:** Anthony Richardson, Franz S. Gruber, David P. Day, Darren Edwards, Irene Georgiou, Zoe C. Johnston, Halimatu Joji, Sarah Martins da Silva, Rachel Myles, Neil R. Norcross, Kevin D. Read, Jason R. Swedlow, Caroline Wilson, Christopher L. R. Barratt, Ian H. Gilbert

**Affiliations:** † Reproductive Medicine Research Group, Division of Systems Medicine, School of Medicine, Ninewells Hospital and Medical School, 3042 University of Dundee, Dundee DD1 9SY, U.K.; ‡ Drug Discovery Unit, Wellcome Centre for Anti-Infectives Research, Division of Biological Chemistry and Drug Discovery, 3042 University of Dundee, Dundee DD1 5EH, U.K.; § Divisions of Computational Biology and Molecular, Cellular and Developmental Biology, School of Life Sciences, 3042 University of Dundee, Dundee DD1 5EH, U.K.; ∥ National Phenotypic Screening Centre, School of Life Sciences, 3042 University of Dundee, Dundee DD1 5EH, U.K.

## Abstract

In this article, we detail our latest findings toward developing a diversified series of potential nonhormonal contraceptive compounds using a phenotypic screening approach against human sperm. Phenotypic screening of nine compound libraries (88,773 compounds in total) was conducted using an in-house automated robotic screening platform, allowing quantification of sperm motility in samples pretreated with the compounds. From these screens, 9 chemical series were identified and investigated in hit expansion programs, with a particular focus on identifying chemical matter that selectively reduces sperm motility without any significant cytotoxicity in somatic cells (HepG2 cells). While there were no clinically progressable leads identified, the study did identify some useful tool compounds for research into the fundamental biology underpinning nonhormonal contraceptive discovery, and a lot was learned about the screening technology, which sets us up for future screening to identify and develop better chemical starting points.

## Graphical abstract

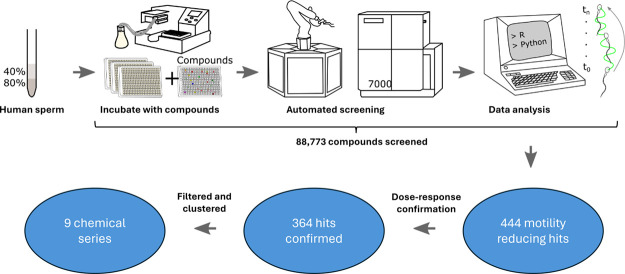

## Introduction

The large number of unwanted pregnancies worldwide (121 million per year^1^) and the associated impact on society are sentinel markers of the need for new methods of contraception. Current birth control options are limited to barrier-based methods, surgical intervention, intrauterine devices, or the female contraceptive pill, which was developed over 60 years ago. As revolutionary as the female contraceptive pill has been, it does cause severe and potentially life-threatening side effects in some women and has compliance issues due to the strict dosing regimen required.^1^ Most of these issues stem from the fact that the contraceptive pill is a hormonal contraceptive with systemic exposure and, therefore, effects throughout the body. In the case of men, the only contraceptive methods are condoms or vasectomy. Therefore, it would be extremely beneficial to develop nonhormonal contraceptives for either women or men. One option to achieve this is to develop small-molecule drugs that impact sperm function. There is clinical data that can be used to inform the effects of sperm dysfunction on human fertility, which makes this a very attractive approach.^2^ There are known biological targets that impact sperm function, some of which are being investigated for nonhormonal contraceptives, such as soluble adenylyl cyclase (sAC),^3^ CatSper,^4,5^ and cyclin-dependent kinase-2 (CDK2).^6^ While a significant amount of work has focused on these targets, no clinical candidates have been developed based on these mechanisms. The first clinical trial of a nonhormonal male contraceptive commenced in December 2023, with YCT-529, which is a retinoic acid receptor alpha (RAR-α) antagonist that has proven very effective in rodent studies.^7,8^ While this is a big breakthrough, the limitation with this drug is related to its mode of action. YCT-529 works via disrupting spermatogenesis, leading to an infertile/subfertile sperm count, but this process takes approximately 2–3 months in humans, which leads to a lag in efficacy and also a lag in reversibility once drug exposure is stopped.

The focus of our research is on using high-throughput phenotypic screening to identify small molecules that impact human sperm function.^9^ In particular, we have been trying to identify small molecules that reduce sperm motility, which is a very common cause of male infertility, so it is an ideal phenotype to target for nonhormonal contraceptives. By targeting disruption of sperm function in mature cells, this opens up the possibility of discovering contraceptive drugs that are dosed to biological males or biological females and, depending on the time to onset of effect, could be used as an on-demand contraceptive or continuous-use contraceptive. As the whole intact human spermatozoon is being assessed, the complete cellular machinery controlling functional process is tested in situ*,* and thus, the data are biologically relevant. Furthermore, the use of human sperm is the clinically relevant target rather than using biological material from an animal species. Target deconvolution of hits has the potential to uncover new elements of sperm cell biology.

We have previously reported that phenotypic screening can identify compounds that enhance or decrease sperm function in an automated high-throughput assay.^10,11^ A preliminary analysis of one repurposing drug library (ReFRAME)^11^ produced limited hit data supporting the concept that we could identify compounds that reduce sperm motility. We therefore set out to explore a bigger compound space. In this study, we utilized the human sperm motility phenotypic screening system to examine nine libraries incorporating a total of almost 90,000 compounds, which is, to the best of our knowledge, the largest reported screen of compounds for their effect on human sperm motility. Libraries consisted of FDA-approved drugs, target-class specific libraries (such as kinase inhibitor libraries), and larger in-house libraries of chemically diverse small molecules, which have been curated to give large coverage within the physicochemical property space of lead-like small molecules. The aim was to identify compounds of interest that can reduce sperm motility and, where appropriate, generate possible start points for a medicinal chemistry program toward a viable contraceptive. A key challenge in contraceptive drug development is safety, a high therapeutic index, owing to the commonality of proteins in sperm and somatic cells. Hence a key driver in the screening process is to identify compounds with good selectivity compared to HepG2 cells.

## Results and Discussion

### Identification of New Compound Series Using HTS

Using our previously established phenotypic human sperm motility screening platform,^11^ we screened a large subset (62,621 compounds) of one of our in-house diversity libraries (National Phenotypic Screening Centre (NPSC) Diversity Set) (Figure 1A,B). Briefly, human sperm are collected, isolated from semen, distributed into 384-well plates with test compounds, time-lapse images of sperm movement are recorded using a high-content imager, and progressive motility parameters are calculated using custom data analysis software. Sperm motility is complicated, and the mechanisms underpinning its regulation are not fully understood. Progressive motility, which is a measurement of the movement of forward progression of sperm, is most closely linked to sperm quality, and the percentage of progressively motile sperm compared to control is therefore the parameter utilized during the work described in this paper.

**Figure 1:**
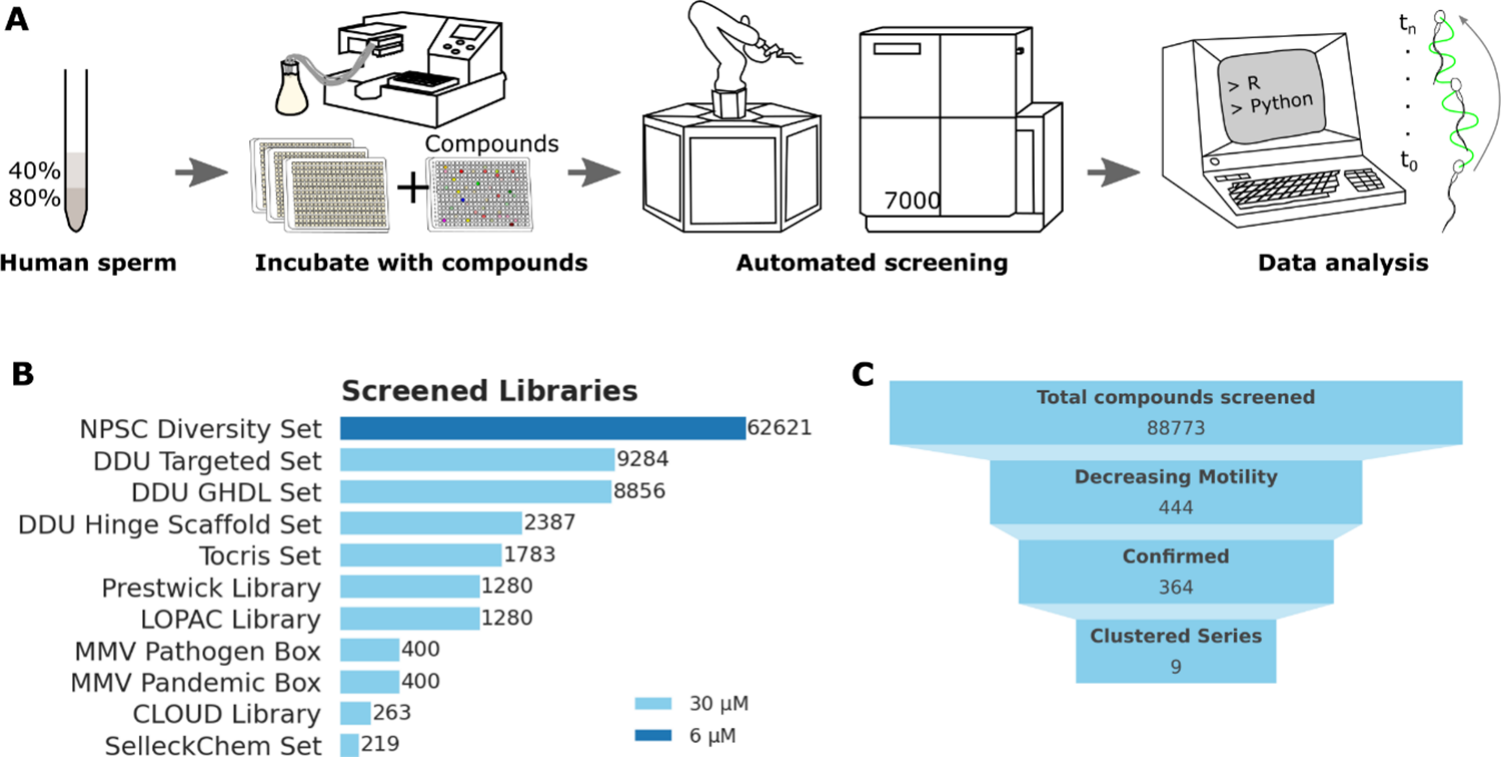
Overview of the screening platform and screening efforts. (A) Donated human sperm samples were prepared using standard differential gradient centrifugation to enrich for high-quality sperm cells used (in 80% fraction) for further experiments. Screening was performed in 384-well format, and sperm cells were dispensed into assay-ready plates containing compounds. A robotic platform was used for automated screening, followed by custom-made data processing and analysis tools. (B) Library size of screened libraries. Color indicates primary screening concentration and incubation time: dark blue 6 μM (10 min incubation with compound), light blue 30 μM (up to 6 h incubation with compound). DDU – Drug Discovery Unit, GHCDL – Global Health Chemical Diversity Library, LOPAC - Library of Pharmacologically Active Compounds, MMV – Medicines for Malaria Venture, CLOUD – CeMM (Centre for Molecular Medicine) Library of Unique Drugs. Funnel graph showing total compounds screened, compounds decreasing motility, confirmed in concentration–response experiments, and selected for potential further investigations.

We detected a few hits (0.1% hit rate), screening at a compound concentration of 6 μM, using a hit cutoff of ≥20% reduction in motility, relative to vehicle control, and 10 min compound incubation time (Figure 2A–C). Therefore, we decided to modify the screening conditions, using a higher concentration of compound (30 μM) and a longer incubation time (a second read-out after 6 h), but still with a cutoff of ≥20% reduction in motility, with the aim of increasing the hit rate to identify more chemical start points. Although these may be less active, having more hits increases the chance of identifying compounds with better physicochemical and other properties, which should be a better starting point for hit development. With these changes to the primary screening conditions, we were able to obtain a higher hit rate, testing several libraries in our platform (Figures 1B and 2A–C). In summary, we screened a total of 88,773 compounds (62,621 with initial screening conditions and 26,152 with relaxed conditions) (Figure 1C), classified 444 compounds as hits (∼0.5% hit rate, Figure 2A), of which 364 confirmed in concentration–response experiments (∼82% of all hits, Figure 2C) and a set of medicinal chemistry filters were used to narrow this down to the compounds of most interest. First, compounds with known toxicophore groups were excluded. Next, the compounds that are flagged as “frequent hitters” (i.e., commonly come up as hits across a range of diverse screens) were excluded, as they are likely to be nonspecific binders. After this, the physicochemical properties of the compounds and the efficiency metrics, such as ligand efficiency (LE) and lipophilic ligand efficiency (LLE), were calculated, and the 11 compounds with the highest efficiency metrics and optimal “lead-like” physicochemical properties were selected and clustered into 9 chemical series for further follow-up (Figure 1C).

**Figure 2:**
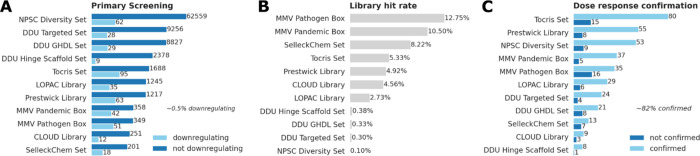
Details of screening efforts. (A) Number of compounds either downregulating or not downregulating. (B) Hit rates of each library. (C) Number of compounds either confirmed or not confirmed in concentration–response experiments.

Compounds selected for further investigation are depicted in Figure 3, alongside information on the library each hit was contained, and its library ID.

**Figure 3:**
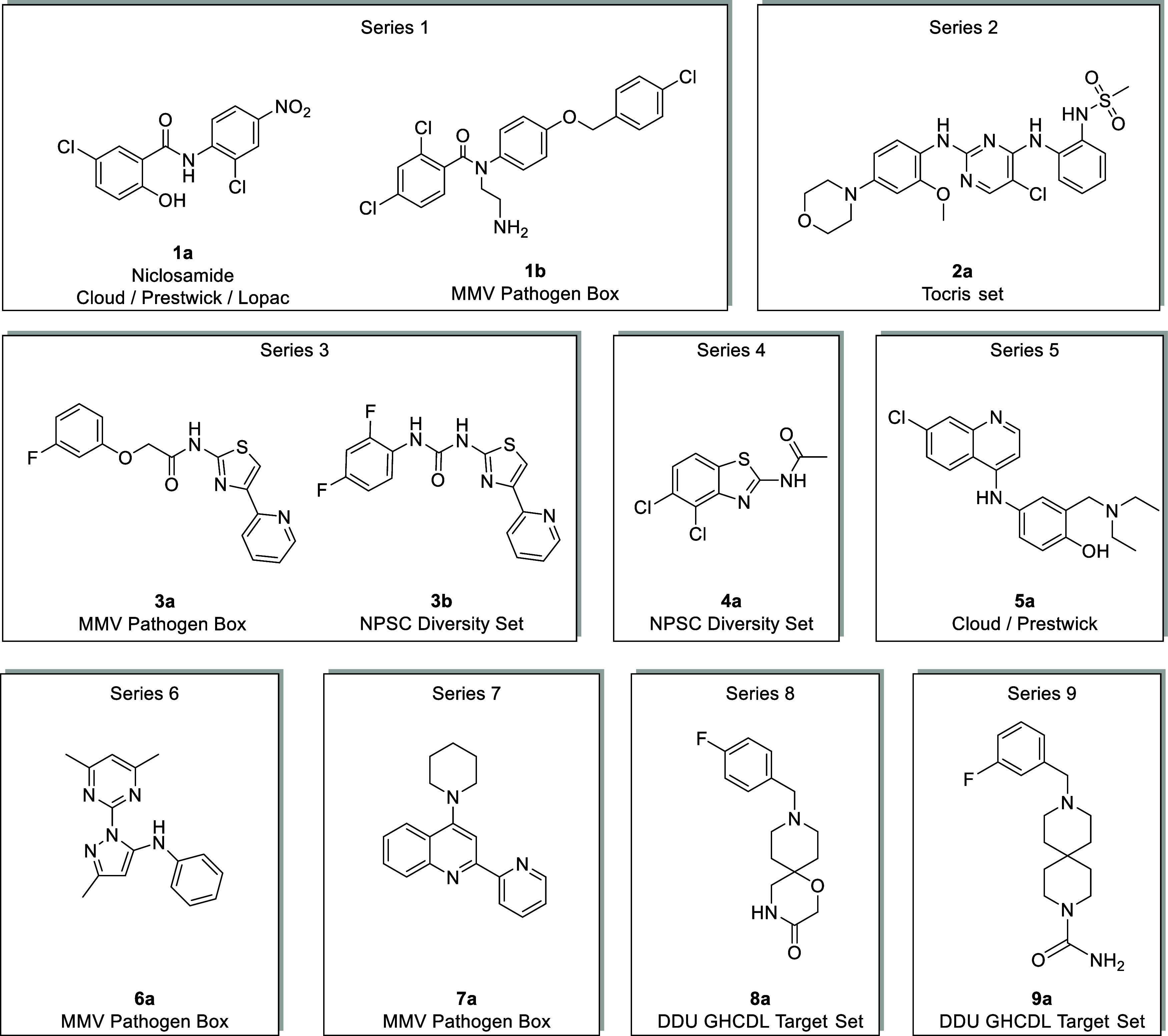
Compounds were selected for further investigation.

### Criteria For Progressing Chemical Series

Our overarching aims for investigating our chemical series and deciding which ones had the potential for further progression were to achieve adequate potency in our motility assay, suitable solubility, and minimal cytotoxicity in HepG2 cells. This third point was often the most challenging, as multiple series had cytotoxicity in HepG2 cells. This observation is perhaps not surprising; many potential targets in sperm cells can also be found in somatic cells. Our cytotoxicity assay is run with an incubation time of 72 h, to give time for gross cytotoxic effects to become apparent.

Our targets for progressing a series into hit-to-lead were to achieve a pEC_50_ > 6 (that is a reduction in sperm motility of 50%, relative to control) after 6 h of incubation in our reduction of sperm motility (RSM) assay, a pEC_50_ < 4.5 in our HepG2 cytotoxicity assay and an aqueous solubility >100 μM. The reason for including an aqueous solubility cutoff in the criteria is that increased solubility is desirable for achieving acceptable oral bioavailability. Although poor solubility can sometimes be addressed during development, this does increase development costs and timelines. As we did not have any information on the target for any of these chemical series, medicinal chemistry efforts were focused on identifying key pharmacophoric functionalities and optimizing potency and physicochemical properties. This was carried out through manipulation of the scaffold appropriate substitutions. Any undesirable functional groups were removed.

### Series 1

The first chemical series identified was based on the two hit compounds **1a** (Niclosamide), from the Centre for Molecular Medicine (CeMM) Library of Unique Drugs (CLOUD), and **1b** (MMV688371), from the Medicines for Malaria Venture (MMV) pathogen box (Table 1). Niclosamide is an approved therapy for the treatment of tapeworm infections and is reported to work by reducing ATP levels in the worms, which may be the result of reducing oxidative phosphorylation and/or increasing ATPase activity.^12^ Compound **1b** has known antiparasitic activity against *Trypanosoma brucei*, although the mechanism of action is unknown.^13^ Both **1a** and **1b** contained 2 aromatic groups linked by a central amide moiety, so they were classed as the same series. Compound **1a** showed good potency in the sperm motility assay but only a small difference (0.5 log units) between the pEC_50_ in the sperm motility assay and the pEC_50_ in the HepG2 cytotoxicity assay and also showed poor aqueous solubility. Compound **1b** showed moderate potency in the sperm motility assay but had a higher pEC_50_ in the HepG2 cytotoxicity assay. The focus on this series was to improve the potency and reduce the cytotoxicity in HepG2 cells. Attempts to probe the structure-activity relationship (SAR) around **1a** were initially focused on changes to the aniline moiety (R^2^). It was found that this ring could tolerate a variety of substituents with little change in potency in the sperm motility assay (**1c–1g**) and the HepG2 cytotoxicity assay. Compound **1c** did show a slight reduction in cytotoxicity and an improvement in aqueous solubility, although cytotoxicity was an ongoing issue. A potential concern is the masked aniline moiety; changing the aniline ring to a 3-pyridyl in an approach to mitigate potential mutagenic risks gave a complete loss of potency in the sperm motility assay (**1h**). The next focus for these analogues was changes to the other aromatic ring (R^1^). Removal of both substituents from this ring gave a complete loss of potency in the sperm motility assay (**1i**). Replacing the 2-OH group with 2-OMe also resulted in a complete loss of potency (**1j**).

**Table 1:**
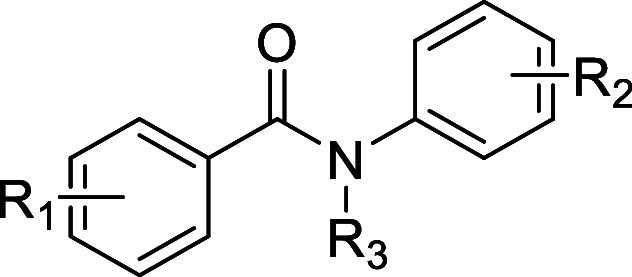

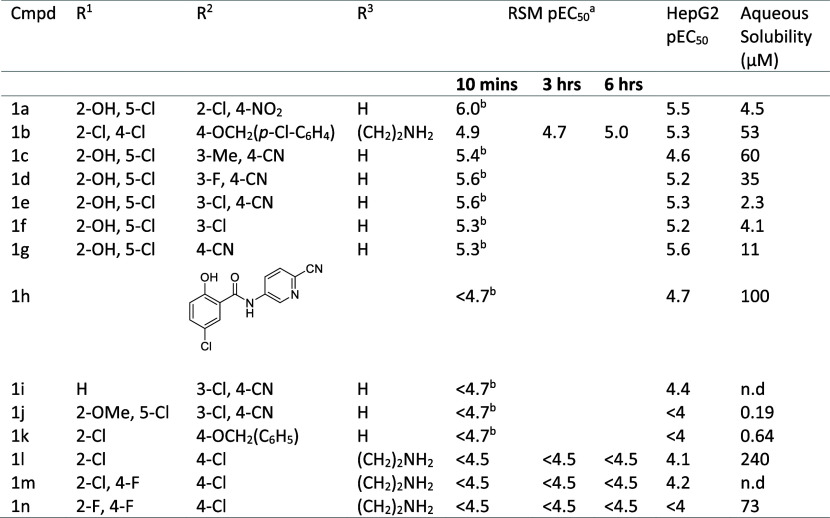
Series 1 SAR

a RSM pEC_50_–pEC_50_ from a concentration–response curve where the signal is reduction of sperm motility, relative to control (note: for each incubation time, if the largest reduction in motility observed relative to control is <70%, the concentration–response curve was not fitted and the pEC_50_ is reported as <X, where X is the negative log of the top concentration of the assay). Motility measurements were taken at 10 min, and 3 and 6 h incubation times.

b Motility is measured only after 10 min of incubation.

Follow-ups to **1b** started with the removal of the aminoethyl group (**1k**), as this was not present in **1a** or analogues of this compound. This compound was inactive in the sperm motility assay, so further compounds included the aminoethyl group. The next step was to try to remove the lipophilic 4-chlorobenzyl ether moiety from R^2^ (**1l–1n**). This also caused a complete loss of potency.

Ultimately, the inability to achieve high potency while removing the cytotoxic liability in this series, combined with the apparent necessity for undesirable functional groups (i.e., aniline and phenol), resulted in the series not being pursued any further.

### Series 2

The second chemical series identified was based on hit compound **2a** from the TocriScreen library (Table 2). **2a** is a reported leucine-rich repeat kinase-2 (LRRK2) inhibitor.^14^ This compound showed good potency in the sperm motility assay but had the same pEC_50_ value in the HepG2 cytotoxicity assay. A variety of changes to R^1^ and R^2^ were investigated; most did not show any improvement in the potency. The most potent compound was **2d**, which showed a 1 log-unit increase in potency in the sperm motility assay after 6 h incubation. However, this compound also showed a 1 log increase in cytotoxicity against HepG2 cells.

**Table 2:**
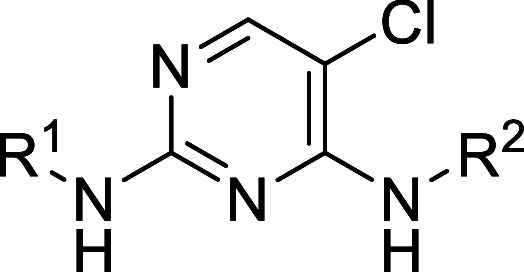

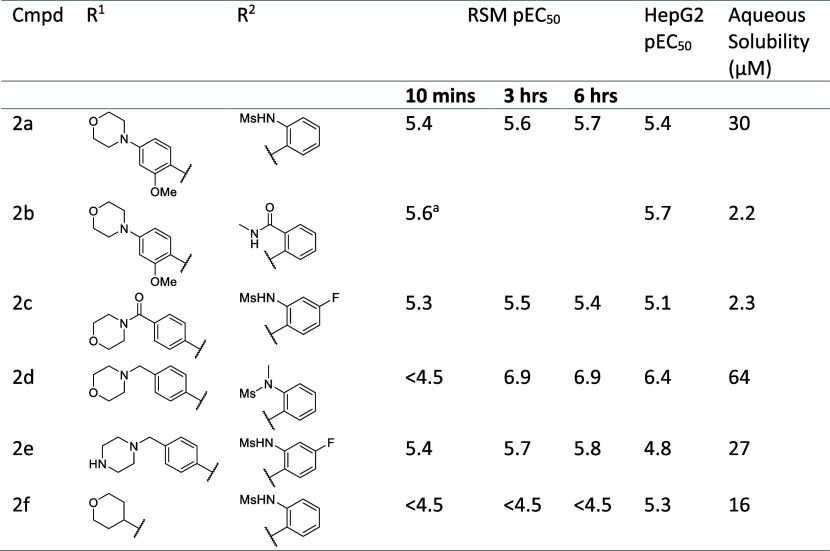
Series 2 SAR

a Motility is measured only after 10 min of incubation for compound **2b**.

The compounds in this series did not show much, if any, window between potency in the sperm motility assay and cytotoxicity in the HepG2 assay. The compounds are likely to be kinase inhibitors, based on structural similarity to known kinase inhibitors that bind to the kinase domain. Inhibition of human kinases may be the source of the cytotoxicity as well as the source of activity. It would be difficult to obtain the exquisite selectivity required for a contraceptive drug with a compound that binds to the active site of a kinase, as many kinases have a high degree of similarity in the kinase domain, and they are also present in somatic cells. Given the challenges of overcoming cytotoxicity and potential issues with kinase selectivity, it was decided to stop the chemistry on the series.

### Series 3

The third chemical series identified was based on hit compound **3a** (MMV676409) from the MMV Pathogen Box and compound **3b** from the NPSC Diversity Set library (Table 3). Compound **3a** is known to inhibit *Mycobacterium tuberculosis* cytidine triphosphate (CTP) synthetase, with some activity against human CTP synthetase 1.^15^ Both compounds showed moderate potency in the sperm motility assay, but compound **3b** showed the same pEC_50_ in the HepG2 cytotoxicity assay. Compound **3c**, which was an analogue of **3a** was inactive in the sperm motility assay. The 2-pyridyl-thiazole moiety has the potential to be a bidentate metal ion chelator. Further, the series contains a masked aminothiazole; these are associated with toxic effects.^16^ Attempts to change the 2-pyridyl ring of **3b** to a 3-pyridyl (**3d**), phenyl (**3e**) or 2,4-difluorophenyl (**3f**) were not tolerated. Replacements of the pyridyl with small aliphatics (**3g** and **3h**) were also not tolerated. To mitigate potential mutagenic issues with the aniline moiety, the difluoroaniline was replaced with a selection of alkylamines. The tetrahydropyran, isopropyl and piperidine amines (**3i–3k**) were all inactive but the isobutylamine (**3l**) was equipotent to **3b**. 2-Aminothiazoles can be prone to forming reactive metabolites via oxidation of the C4–C5 double bond to an epoxide. To mitigate this, we employed two strategies. First, the 5-methylthiazole analogue (**3m**) was made to sterically block against oxidation. This compound was more potent and marginally less cytotoxic than **3b** but still has less than 1 log-unit difference between the sperm motility assay pEC_50_ and the pEC_50_ from the HepG2 cytotoxicity assay. Second, the oxazole analogue (**3n**) was made, which is far less prone to oxidation. This compound was found to be inactive in the sperm motility assay.

**Table 3:**
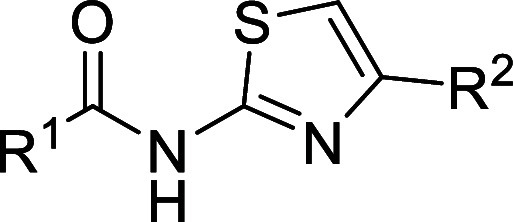

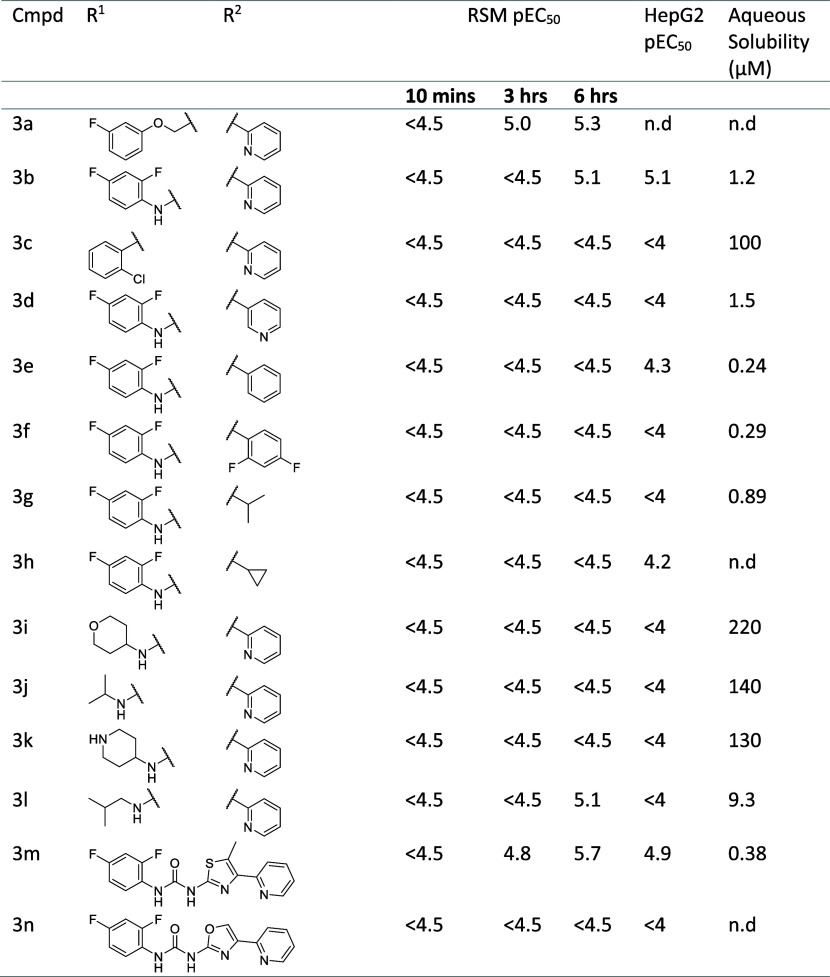
Series 3 SAR

Due to a lack of significant potency boosts and high levels of HepG2 cytotoxicity, this series was stopped.

### Series 4

The fourth chemical series identified was based on hit compound **4a** from the NPSC Diversity Set library (Table 4). This compound showed moderate potency in the sperm motility assay and approximately a 1 log-unit difference between potency in the sperm motility assay and HepG2 cytotoxicity. The initial attempts to probe the SARs on this series revolved around changes to the amide moiety (**4b**–**4h**). It appeared that this part of the molecule was fairly tolerant to changes, with most aliphatic or aromatic amides being equipotent, although all of the changes resulted in worse solubility. Removal of the amide to leave the primary amine (**4b**) or changing the amide to the primary urea (**4h**) gave a loss in potency. Some of the changes, such as **4e** and **4g**, maintained potency and slightly reduced the HepG2 cytotoxicity.

**Table 4:**
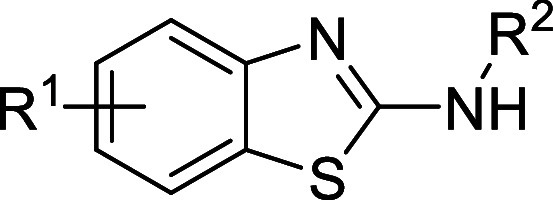

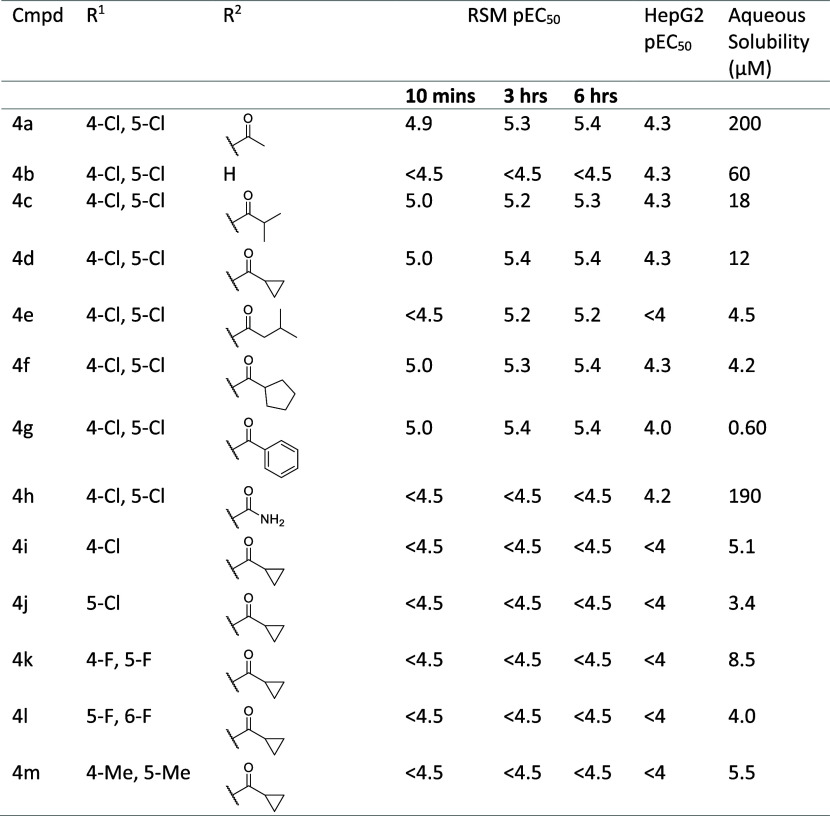
Series 4 SAR

The next area of the molecule that was investigated was the substitution around the benzo ring, and it was found that removal of one of the chlorines (**4i** and **4j**) or changing them to fluorines (**4k**) or methyls (**4m**) gave a complete loss in potency in the sperm motility assay.

Given the flat SAR around the amide and the lack of tolerance to changes around the benzo ring substituents, combined with the fact that this series showed generally poor metabolic stability in microsomes (data not reported), the series was stopped.

### Series 5

The fifth chemical series identified was based on amodiaquine (**5a**), a medication employed for the treatment of malaria, present in the CLOUD/Prestwick libraries (Table 5). The initial hit showed good potency in the sperm motility assay and a moderate window between the potency and cytotoxicity in HepG2 cells. In total, 47 analogues were accessed in attempts to improve on the initial hit, with selected results shown in Table 5. Initial exploration focused on the benzylic amine moiety. It was found that changes to the amine were well tolerated, with pyrrolidine (**5b**) and piperazine (**5c**) both being potent. Changing the amine to the amide (**5d**) was not tolerated, but a complete removal of the amine-containing group (**5e**) was equipotent to the initial hit. The main concern for this series was the 4-aminophenol moiety, which is a potential toxicophore; therefore, the phenyl ring was saturated to give **5f**, which was inactive in the sperm motility assay. As an alternative approach, the OH was changed to OMe (**5g**) or F (**5h**), and these also lost potency. Next, the 2-pyridyl analogues **5i** and **5j** were made, and these also lost potency. To explore alternative H-bond donors in place of the phenol, indole **5k** was made and was also inactive.

**Table 5:**
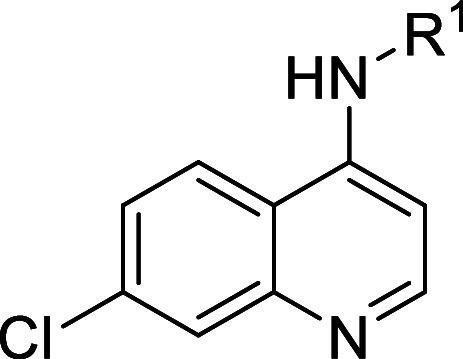

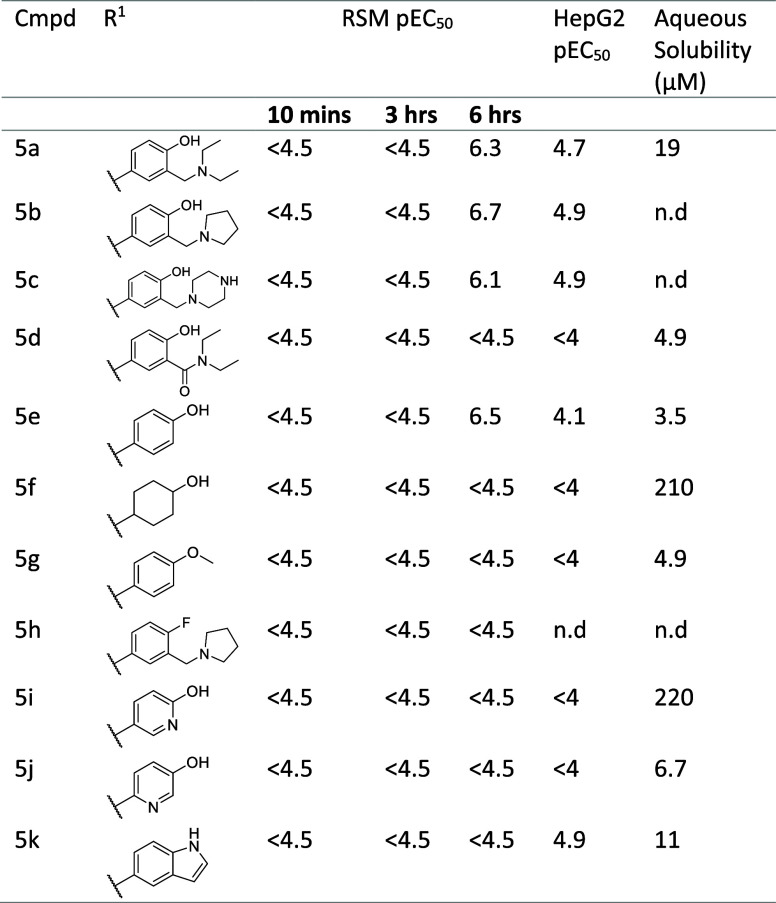
Series 5 SAR

Given the apparent necessity for the 4-aminophenol moiety, which is a known toxicophore, this series was stopped.

### Series 6

The sixth chemical series identified was based on hit compound **6a** (MMV062221) from the MMV Pathogen box (Table 6), which is a reported antimalarial.^17^ This compound showed good potency in the sperm motility assay. The close analogue **6b**, with an additional Me substituent on the phenyl ring, was found to be equipotent and not cytotoxic against HepG2 cells. A handful of structurally similar analogues from DDU libraries were screened and found to be inactive; therefore, the series was not pursued any further.

**Table 6:**
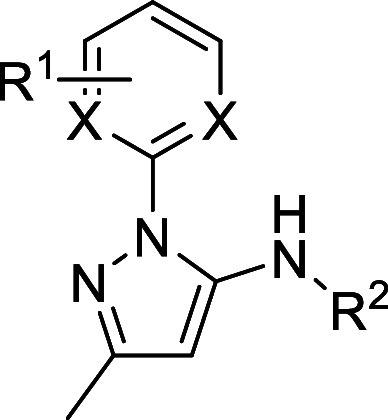

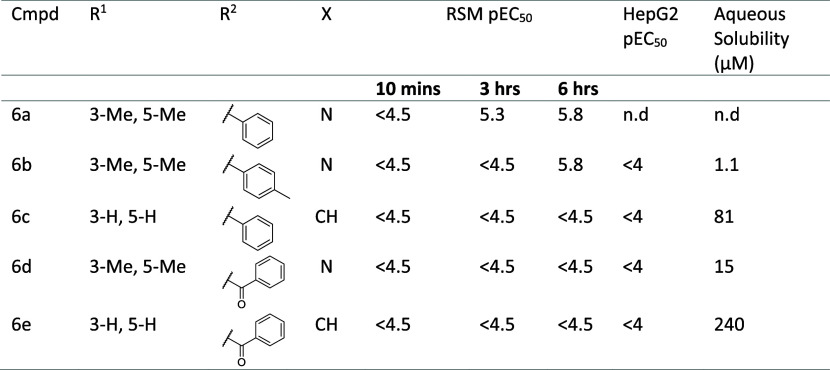
Series 6 SAR

### Series 7

The seventh chemical series identified was based on hit compound **7a** (MMV560185) from the MMV Pathogen box (Table 7), which is reported to inhibit methionine aminopeptidase-1 (MetAP1).^18^ This compound showed good potency in the sperm motility assay and was not cytotoxic against HepG2 cells. The 2,2′-bipyridyl setup has the potential to act as a metal chelator, so initial chemistry efforts were focused around changing the 2-pyridyl group. Phenyl (**7b**) and 3-pyridyl (**7c**) were inactive, as were aliphatic replacements (**7d–7g**), so the series was stopped, as the compound series was almost certainly acting as a metal ion chelator, for which it would prove to be extremely challenging to obtain sufficient selectivity.

**Table 7:**
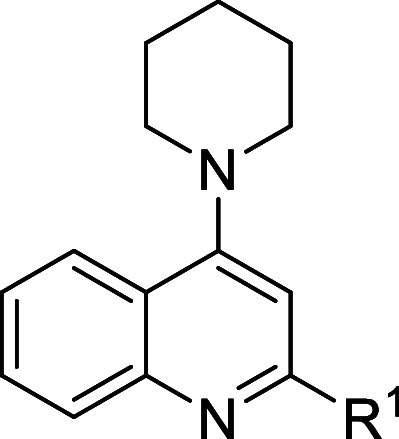

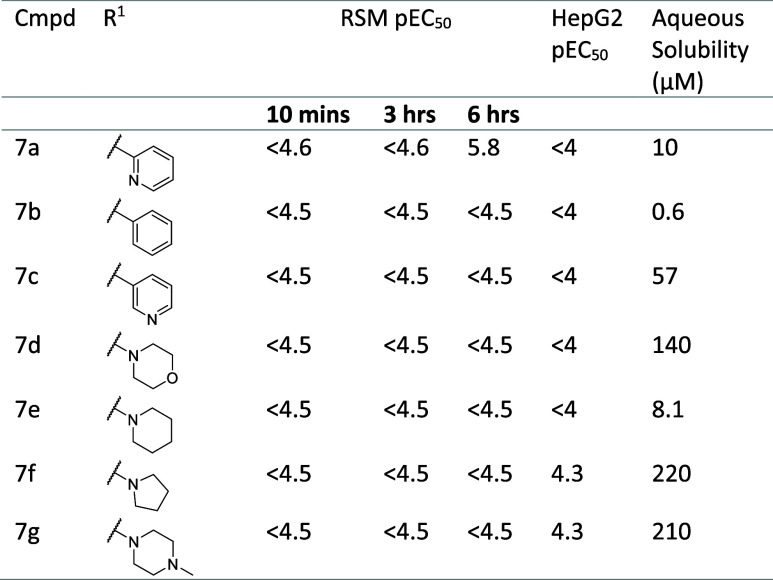
Series 7 SAR

### Series 8

The eighth chemical series identified was based on hit compound **8a** from the Drug Discovery Unit Global Health Chemical Diversity Library (DDU GHCDL) Target set (Table 8). This compound showed good potency in the sperm motility assay and was not cytotoxic against HepG2 cells. An expansion screen of similar compounds in the DDU GHCDL Expansion set gave a few compounds of interest. Heterocycles **8b–8e** were tolerated, but heterocycles **8f** and **8g**, which contained heteroatoms adjacent to the attachment point, were not tolerated. A further exploration of this vector was attempted via plate chemistry, with the crude reaction mixtures screened for potency in a single-point screen. A selection of aliphatic and aromatic groups, with and without the methylene spacer or with branching off the methylene, was included in the screen, and they all came back inactive. Given the good potency for compounds **8a–8e**, this result seemed surprising, so compound **8h**, which was in the plate chemistry screen, was resynthesized, purified, and tested in concentration–response and still found to be inactive. It was concluded that this area of the molecule was not as tolerant of changes as initially thought from the expansion screen.

**Table 8:**
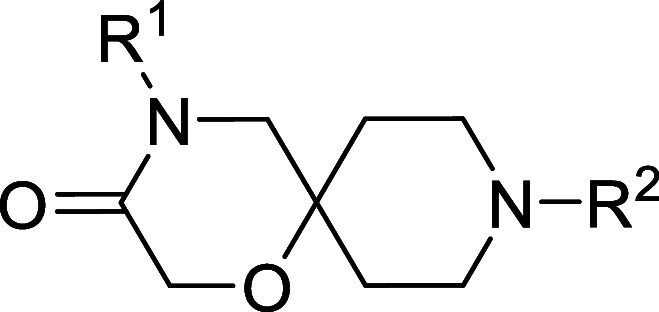

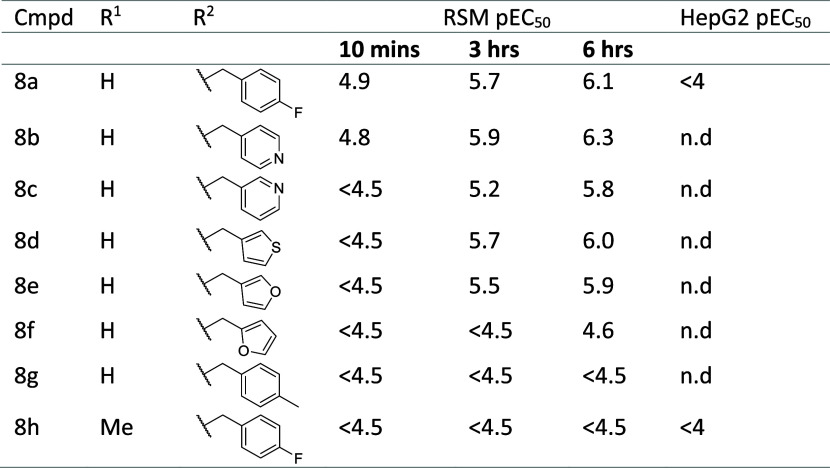
Series 8 SAR

From the expansion screen, it was discovered that the carbons of the spirocycle were not tolerant to substitution and removals/changes to the heteroatoms in the spirocycle were not tolerated. The only remaining vector was N-substitution from the spirocyclic lactam. To this end, the N-Me analogue (**8i**) was made and found to lose all activity. It was decided that the NH must be necessary for potency.

Given the lack of opportunities to grow the molecule and develop any SAR, the series was terminated.

### Series 9

The ninth chemical series identified was based on hit compound **9a** from the DDU GHCDL Target set (Table 9). This compound showed moderate potency in the sperm motility assay and was not cytotoxic with HepG2 cells. An expansion screen of similar compounds in the DDU GHCDL Expansion set did not identify any additional active compounds. The closest analogue to **9a** in the expansion screen was compound **9b**, which has the acetamide in place of the primary urea. This was inactive, suggesting that an H-bond donor is probably necessary in this area. From the expansion set, it was not clear if both H-bond donors of the NH_2_ were necessary, as no secondary ureas were part of the expansion screen. In order to check this, compound **9c** was synthesized and tested and this was also found to be inactive, suggesting either both H-bond acceptors were necessary or there is limited space around the urea. The compound with the urea removed (**9d**) was also inactive. Even a minor change to the aromatic substitution, moving the 3-F to 4-F (**9e**), lost all potency.

**Table 9:**
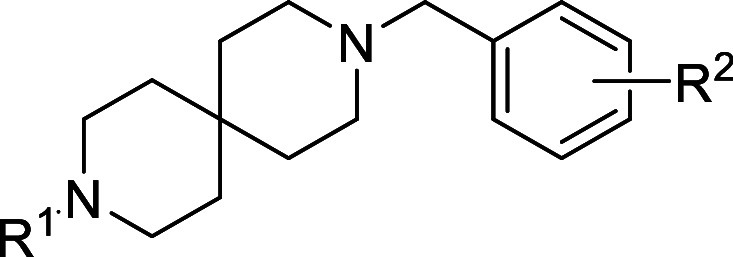

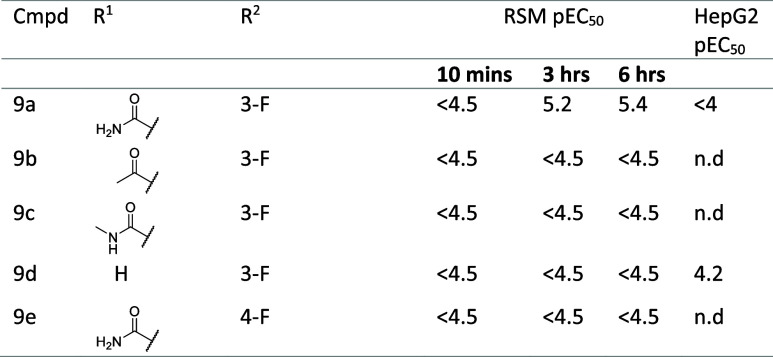
Series 9 SAR

Given the loss of potency for all analogues of the initial hit, this series was terminated.

## Conclusions

Through our phenotypic screening, we have identified numerous compound series that reduce sperm motility. However, most of these have issues with cytotoxicity in HepG2 cells. Other compounds have tight or flat SARs, precluding further development. Compounds **8a** and **9a** could be used as tool compounds, given that they exhibited good potency in the sperm motility assay and had no measurable cytotoxicity in HepG2 cells. Interestingly, where compounds were active, there appeared to be some time-dependent effect on activity, with a reduction in sperm motility increasing with an increased time of exposure. We are not sure of the reasons for this, given the diversity of chemotypes examined. There would be benefits to identifying compounds with a more rapid onset of action for use as on-demand contraceptives for male or female dosing, and the screening technology would allow these compounds to be identified. Some of the compounds from series 1 and 2 showed similar potency after 10 min of incubation with sperm cells as they did after 6 h of incubation, but these compounds were also found to be cytotoxic in our HepG2 assay, so it is possible that they are just fast-acting cytotoxins.

It is not surprising that identifying compounds that adversely affect sperm motility without general cytotoxicity in somatic cells is challenging. Sperm cells contain enzymes and receptors that significantly overlap with those found in somatic cells. Therefore, obtaining selective compounds is not straightforward. The ideal scenario would be to identify compounds that target enzymes or receptors that are present in sperm cells but not in somatic cells or where the sperm cells have a different isoform to somatic cells. Failing that, identifying compounds that act on enzymes or receptors that have a critical role in sperm cells but not in somatic cells would be a viable option.

Given the relatively poorly understood biology of human spermatozoa, the selection of a molecular target is challenging. We have found in other therapeutic areas, where there are few validated drug targets, that phenotypic screening in combination with target deconvolution is a powerful way to identify and validate drug targets.^19,20^ Once targets are identified, it should be possible to identify progressable and developable chemical matter. To follow this strategy, we will have to screen a more diverse range of chemistry, covering a wider range of chemical space, to identify sperm selective hits.

Although none of the chemical series disclosed were deemed to be progressable, the project allowed us to develop and validate our screening platform and gain a better understanding of sperm biology, which will be utilized in future screening programs to identify better starting points.

## Materials and Methods

### Experimental Design

We used an HTS screening platform to assess the motility of live human spermatozoa. The platform and its development are described in detail in Gruber et al.^10,11^ and summarized below in brief. The platform was used to screen compound libraries specifically focusing on reduction in motility. Interesting compounds were examined in further detail as possible starting points for a medicinal chemistry program. The experimental design is illustrated in Figure 1.

### Selection and Preparation of Spermatozoa

Full details of the HTS system, and its development, are discussed by Gruber et al.^10,11^ Semen samples were obtained from volunteer donors. Written consent was obtained from each donor in accordance with the Human Fertilization and Embryology Authority (HFEA) Code of Practice (version 8) and local ethical approval (Tayside Committee of Medical Research Ethics B, 13/ES/0091 and University of Dundee, SMED REC 20/45).

Donors were local to the Dundee area, over the age of 18, and had no known fertility problems and normal sperm concentration, motility and semen characteristics according to WHO 2021 criteria.^21^ Special category personal data for donors, such as ethnic origin, was not recorded in accordance with ethical approval and UK GDPR. Samples were obtained by masturbation, after sexual abstinence of 2–5 days, and delivered to the research laboratory within one h of production. Samples were allowed to liquefy at 37 °C for 15–30 min prior to preparation by discontinuous density gradient centrifugation (DGC). Gradients were prepared using Percoll (Sigma-Aldrich, UK) diluted to 80% and 40% with media that does not support capacitation (Minimal Essential Medium Eagle Sigma M3024, supplemented with HEPES, Sodium lactate, and Sodium pyruvate to achieve concentrations previously described).^22^ A minimum of 3 prepared donor samples were routinely pooled to reduce donor-to-donor variability for primary screening assays. Pooled samples were incubated for 3 h at 37 °C under noncapacitating conditions.

### High-Throughput Screening System

In brief, screening batches of cells were transferred to a robotic platform (HighRes Biosolutions Inc.) and maintained during the screen at 37 °C at 5% CO_2_. Assay-ready 384-well plates containing compounds were prepared prior to the screen and filled with approximately 10,000 spermatozoa (20 μL) per well using a liquid handling system (MultiDrop Combi; ThermoFisher). These plates were incubated for 10 min prior to imaging. The HTS system utilized a Yokogawa CV7000 Cell Voyager high-throughput microscope to record time-lapse images from 2 positions in each well. An adaptation of a tracking algorithm, Trackpy v0.4.1 ^23^ was utilized to track individual spermatozoa within each well and obtain kinematic parameters. Within the compound-test plates, DMSO was used as the vehicle control.

### Initial Libraries Screened

1. The Pathogen box (Medicines for Malaria Venture [MMV] generously provided by MMV, https://www.mmv.org/mmv-open/pathogen-box/about-pathogen-box. This is a small repurposing library assembled to screen against rare and neglected tropical diseases containing ∼400 diverse, drug-like molecules with demonstrated biological activity against different pathogens.
2. The Pandemic box (MMV generously provided by MMV, https://www.mmv.org/mmv-open/pandemic-response-box/about-pandemic-response-box). This is a small library with 400 drug-like molecules with activity against bacteria, viruses, and fungi.
3. The CeMM Library of Unique Drugs (CLOUD) purchased from Enamine (https://enamine.net/hit-finding/compound-collections/bioreference-compounds/the-comprehensive-drug-collection-cloud) is a set of 263 small molecules representing the target and chemical space of FDA-approved drugs that has been used for drug repurposing.
4. Tocris Set 1783 compounds (Tocris, Bristol, United Kingdom, https://www.tocris.com/products/tocriscreen-plus_5840), comprising 1280 biologically active small-molecule compounds originally in the Tocris Screenplus library and 503 biologically active small-molecule compounds, which are now part of the Tocriscreen 2.0 libraries.
5. LOPAC1280LOPAC (Library of Pharmacologically Active Compounds https://www.sigmaaldrich.com/life-science/cell-biology/bioactive-small-molecules/lopac1280-navigator.html). These compose a biologically annotated collection of inhibitors, receptor ligands, pharma-developed tools, and approved drugs which impacts many signaling pathways and covers all major drug target classes (1280 compounds).
6. Prestwick Chemical Library (http://www.prestwickchemical.com/libraries-screening-lib-pcl.html), comprising 1280 off-patent drugs with high chemical and pharmacological diversity as well as known bioavailability and safety in humans.
7. DDU NPSC Diversity Set 62,621 compounds. Subset of larger in-house NPSC diversity Set.
8. DDU Targeted Set 9284 compounds. Subset of larger in-house DDU library.
9. DDU Kinase library 2387 compounds. A library containing small molecules designed to bind ATP pockets of Kinases (“hinge binder”).
10. DDU GHDL (Gates Global Health Chemical Diversity) library targeting set 8856 compounds. Subset of larger in-house DDU library. Compounds enriched for sp3 character.
11. SelleckChem Set 219 compounds. Small custom assembled library with compounds related to kinases, GPCRs, ion channels, and proteases (https://www.selleckchem.com/screening-libraries.html).

### Data Normalization and Primary Screening

All steps were performed as previously described.^10,11^ In summary, data from every compound well were normalized to those from in-plate DMSO controls (wells containing the same amount of DMSO as compound wells). Two positions were recorded in every well, and the average of those positions was used for calculating % of control (median value/DMSO median) × 100. Each plate contained 16 DMSO control wells. Median curvilinear velocity (VCL) was used as the primary readout for the NPSC Diversity Set screened at 6 μM, and percentage progressive motility (PM) was used for all other libraries screened. Hits from the primary screening were chosen based on the % change compared to the control. In these experiments, an RSM of ≥20% compared to negative controls (DMSO) was chosen as a cutoff for potential progression.

### Identification and Criteria of a Compound for Hit Progression

In a screening program of this scale, a significant number of compounds are likely to have a negative effect on sperm motility, but a number will show general toxicity. Furthermore, many will not be tractable for medicinal chemistry. Therefore, any hit confirmed in concentration–response analysis (tested on two independent donor pools) was assessed for any undesired chemical motifs before further investigation. Concentration-response experiments were performed using 8-point, 3-fold curves (30 μM top concentration), with a minimum of 2 donor pools (biological replicates), each screened in duplicate (technical replicates). Data were analyzed with custom R or Python scripts.

Hit confirmation was defined as a maximum RSM of >70% compared to controls after 6 h of incubation in a concentration-response screen, with an EC_50_ of <1 μM, and the chemical material was checked for identity and purity (>95%).

### Hit Analysis

Chemical space was visualized by generating RDK fingerprints using RDKit (default parameters) and UMAP^20^ using the following custom parameters (metric = “jaccard,” n_neighbors = 250, min_dist = 0.1). Physicochemical properties were calculated using RDKit (http://www.rdkit.org) in Python.

### Hit-to-Lead (HTL) Criteria

The following criteria were adopted: RSMs pEC_50_ > 6 and >70% RSM at 6 h incubation; HepG2 pEC_50_ < 4.52, (>100-fold window between HepG2 EC_50_ and RSM EC_50_); aqueous. solubility >100 μM; mouse microsomes/hepatocytes <5 mL/min/g; permeability PAMPA > 10 nm/s, MDCK > 100 nm/s; and plasma protein binding (<95%).

### Aqueous Solubility

As previously described.^24^

### HepG2 Assay

Compound dilution curves were plated directly using a Labcyte Echo 550 acoustic dispenser (125 nL) in 384-well white clear-bottom plates (Greiner). HepG2 cells (ATCCHepG2 HB-8065) were cultured in minimum essential medium (supplemented with glutamax) with 10% FCS and plated (25 μL) using a WellMate dispenser (1 × 105 per well) and incubated for 72 h. Doxorubicin was used as a positive control drug. Resazurin was then added to each well at a final concentration of 45 μM, and fluorescence was measured using a PHERAstar LS (BMG Labtech) after 4 h of further incubation (excitation of 528 nm and emission of 590 nm). Raw data were normalized to controls and expressed as % growth. IC_50_ was defined as the compound concentration that resulted in 50% inhibition.

### General Chemistry Experimental

All reactions were performed by using standard laboratory equipment and glassware. Chemicals and solvents were purchased from the Aldrich Chemical Co., Fluka, ABCR, VWR, Acros, Fluorochem and Alfa Aesar and were used as received. Analytical thin-layer chromatography (TLC) was performed on precoated TLC plates (layer 0.20 mm silica gel 60 with fluorescent indicator UV 254, from Merck). Developed plates were air-dried and analyzed under a UV lamp (UV 254/365 nm). Flash column chromatography was performed using prepacked silica gel cartridges (230–400 mesh, 40–63 μm, from SiliCycle) using a Teledyne ISCO Combiflash Companion, or Combiflash Retrieve. ^1^H and ^13^C NMR spectra were recorded on either a Bruker Avance DPX 500 spectrometer (^1^H at 500 MHz and ^13^C at 125 MHz) or a Bruker Avance DPX 400 spectrometer (^1^H at 400 MHz and ^13^C at 101 MHz). Chemical shifts (d) are expressed in ppm recorded using the residual solvent as the internal reference in all cases. Signal splitting patterns are described as singlet (s), doublet (d), triplet (t), quartet (q), multiplet (m), broad (br), or a combination thereof. Coupling constants (*J*) are quoted to the nearest 0.1 Hz. LCMS analysis was conducted using either an Agilent 6130 ESI Mass Spectrometer, Agilent 1200 HPLC with diode array detector (HPLC chromatographic separations were conducted using a Waters XBridge C18 column, 2.1 × 30 mm, 2.5 μm particle size; mobile phase, water/acetonitrile +0.1% Ammonia), or an HPLC/MS: Shimadzu LC-MS 2020 with photodiode array detector (Signal settings: APCI and ESI Positive/Negative 100–1000 *m*/*z*, Column: THERMO Hypersil Gold 1.9 μm 50 × 2.1 mm column, Eluent: A1 0.1% FA in H20, B1 0.1% Fa in MeCN, Detection signal: 254 nm, Injection: 1 μL standard injection, Column Temp: 40 °C, Flow: 0.8 mL/min, Gradient: 5–95% B, 3 min).

1-(4,6-dimethylpyrimidin-2-yl)-3-methyl-N-phenyl-1H-pyrazol-5-amine **6a** was screened from the MMV Pathogen/Pandemic Box and NMR/*m*/*z* analysis was not conducted. All other hit compounds from series **1–9** with their corresponding analytical data are reported below.

#### Niclosamide **1a**

^1^H NMR (400 MHz, DMSO-d^6^) δ 11.53 (1H, bs), 8.82 (1H, d, *J* = 9.2 Hz), 8.45 (1H, d, *J* = 2.6 Hz), 8.30 (1H, dd, *J* = 9.2, 2.7 Hz), 7.97 (1H, d, *J* = 2.8 Hz), 7.53 (1H, dd, *J* = 8.8, 2.8 Hz), 7.09 (1H, d, *J* = 8.8 Hz), 5.75 (1H, s). ^13^C NMR (101 MHz, DMSO-d^6^) δ: 162.6, 155.3, 142.6, 141.2, 133.9, 130.0, 124.7, 123.8, 123.6, 122.4, 120.8, 119.4, 119.2.

#### *N*-(2-Aminoethyl)-2,4-dichloro-*N*-(4-((4-chlorobenzyl)­oxy)­phenyl)­benzamide **1b**

^1^H NMR (400 MHz, CDCl_3_) δ 7.37–7.29 (4H, m), 7.23 (1H, d, *J* = 1.5 Hz), 7.07–7.02 (4H, m), 6.76 (2H, d, *J* = 8.9 Hz), 4.93 (2H, s), 3.95 (2H, t, *J* = 6.2 Hz), 2.94 (2H, t, *J* = 6.5 Hz), 1.40 (2H, bs). ^13^C NMR (101 MHz, CDCl_3_) δ: 167.9, 157.8, 135.6, 135.0, 134.9, 134.8, 134.2, 131.5, 129.51, 129.45, 129.1, 129.0, 128.9, 126.8, 115.5, 69.5, 52.5, 40.1. HRMS (ES+): *m*/*z* [M + H]^+^ calcd for C_22_H_19_Cl_3_N_2_O_2_ [M + H]^+^ 449.0585, found 449.0573.

#### *N*-(2-((5-Chloro-2-((2-methoxy-4-morpholinophenyl)­amino)­pyrimidin-4-yl)­amino)­phenyl)­methanesulfonamide **2a** ^22^

^1^H NMR (500 MHz, CDCl_3_) δ 8.08 (s, 1H), 7.76 (1H, d, *J* = 8.8 Hz), 7.69–7.65 (1H, m), 7.55–7.50 (1H, m), 7.39 (1H, s), 7.35–7.29 (2H, m), 7.25 (1H, s), 6.94 (1H, s), 6.47 (1H, d, *J* = 2.5 Hz), 6.31 (1H, dd, *J* = 8.8, 2.6 Hz), 3.88–3.84 (4H, m), 3.83 (3H, s), 3.11–3.06 (4H, m), 2.90 (3H, s). ^13^C NMR (125 MHz, CDCl_3_) δ: 158.1, 156.7, 155.3, 149.6, 147.7, 133.0, 130.7, 127.6, 127.0, 126.5, 125.9, 122.1, 120.6, 107.8, 104.8, 100.2, 67.1, 55.8, 50.5, 39.8. HRMS (ES+): *m*/*z* [M + H]^+^ calcd for C_22_H_25_ClN_6_O_4_S [M + H]^+^ 505.1419, found 505.1425.

#### 2-(Phenoxymethyl)-*N*-(4-(pyridin-2-yl)­thiazol-2-yl)­benzamide **3a** ^23^

^1^H NMR (CDCl_3_, 400 MHz) 9.76 (1H, bs), 8.66–8.64 (1H, m), 7.92 (1H, d, *J* = 7.9 Hz), 7.75 (1H, td, *J* = 7.7, 1.8 Hz), 7.72 (1H, s), 7.32 (1H, td, *J* = 8.3, 6.7 Hz), 7.23 (1H, ddd, *J* = 7.5, 4.8, 1.1 Hz), 6.82–6.78 (2H, m), 6.75 (1H, dt, *J* = 10.2, 2.4 Hz), 4.73 (2H, s). ^19^F NMR (471 MHz, DMSO-d^6^) δ −110.07 – −110.13 (m). ^13^C NMR (CDCl_3_, 100 MHz) 166.8, 163.8 (d, *J* = 247.4 Hz), 158.0 (d, *J* = 10.8 Hz), 156.6, 152.4, 150.3, 149.9, 137.0, 131.1 (d, *J* = 9.8 Hz), 123.0, 120.8, 112.5, 110.5 (d, *J* = 2.9 Hz), 109.9 (d, J = 21.2 Hz), 103.2 (d, *J* = 25.4 Hz), 67.2. HRMS (ES+): *m*/*z* [M + H]^+^ calcd for C_16_H_12_FN_3_O_2_S [M + H]^+^ 330.0713, found 330.0711.

#### 1-(2,4-Difluorophenyl)-3-(4-(pyridin-2-yl)­thiazol-2-yl)­urea **3b**

^1^H NMR (400 MHz, DMSO-d^6^) δ 10.97 (1H, s), 8.92 (1H, bs), 8.60 (1H, d, *J* = 4.5 Hz), 8.10–8.06 (1H, m), 7.95 (1H, d, *J* = 7.7 Hz), 7.89 (1H, t, *J* = 7.8 Hz), 7.77 (1H, s), 7.35 (2H, td, *J* = 11.8. 5.5 Hz), 7.10 (1H, t, *J* = 8.0 Hz). ^19^F NMR (471 MHz, DMSO-d^6^) δ −116.6 - −116.1 (m), −124.1. ^13^C NMR (101 MHz, DMSO-d^6^) δ 159.3 (s), 158.7 (d, *J* = 11.8 Hz), 156.3 (d, *J* = 11.5 Hz), 153.8 (d, *J* = 12.4 Hz), 151.4 (dd, *J* = 14.6, 9.8 Hz), 149.0 (s), 148.5 (s), 137.7 (s), 123.0 (d, *J* = 3.4 Hz), 122.9 (s), 122.5 (d, *J* = 9.1 Hz), 120.2 (s), 111.4 (d, *J* = 3.4 Hz), 111.1 (d, *J* = 3.2 Hz), 103.9 (dd, *J* = 26.9, 23.6 Hz). HRMS (ES+): *m*/*z* [M + H]^+^ calcd for C_15_H_10_F_2_N_4_OS [M + H]^+^ 333.0622, found 333.0618.

#### *N*-(4,5-Dichlorobenzo­[d]­thiazol-2-yl)­acetamide **4a**

^1^H NMR (400 MHz, DMSO-d^6^) δ 9.84 (1H, s), 7.95 (1H, d, *J* = 8.8 Hz), 7.78 (1H, d, *J* = 8.8 Hz), 2.15 (3H, s). ^13^C NMR (101 MHz, DMSO-d^6^) δ: 169.0, 138.4, 132.8, 130.8, 125.9, 124.6, 120.8, 110.2, 23.5. HRMS (ES+): *m*/*z* [M + H]^+^ calcd for C_9_H_6_Cl_2_N_2_OS [M + H]^+^ 260.9651, found 260.9656.

#### 4-((7-Chloroquinolin-4-yl)­amino)-2-((diethylamino)­methyl)­phenol **5a**

^1^H NMR (400 MHz, DMSO-d^6^) δ 8.85 (1H, s), 8.40 (1H, d, *J* = 9.1 Hz), 8.36 (1H, d, *J* = 5.4 Hz), 7.84 (1H, d, *J* = 2.2 Hz), 7.51 (1H, dd, *J* = 9.0, 2.2 Hz), 7.08–7.06 (2H, m), 6.78 (1H, d, *J* = 8.2 Hz), 6.56 (1H, d, *J* = 5.4 Hz), 3.74 (2H, s), 2.58 (4H, q, *J* = 7.1 Hz), 1.04 (6H, t, *J* = 7.1 Hz).*OH/NH signal not observed. ^13^C NMR (101 MHz, DMSO-d^6^) δ: 161.7, 155.0, 151.8, 149.5, 133.6, 130.4, 127.5, 125.3, 124.6, 124.5, 124.2, 124.0, 117.7, 116.0, 100.5, 54.8, 45.9, 11.1. HRMS (ES+): *m*/*z* [M + H]^+^ calcd for C_20_H_22_ClN_3_O [M + H]^+^ 356.1530, found 356.1547.

#### 1-(4,6-Dimethylpyrimidin-2-yl)-3-methyl-*N*-(*p*-tolyl)-1*H*-pyrazol-5-amine **6b**

^1^H NMR (400 MHz, DMSO) δ 10.32 (1H, s), 7.17–7.09 (5H, m), 5.95 (1H, s), 2.51 (6H, s), 2.26 (3H, s), 2.18 (3H, s). ^13^C NMR (100 MHz, DMSO-d^6^) δ: 168.1, 156.8, 150.4, 145.8, 138.4, 130.2, 129.8, 117.3, 116.3, 90.0, 23.4, 20.2, 14.0. HRMS (ES+): *m*/*z* [M + H]^+^ calcd for C_17_H_19_N_5_ [M + H]^+^ 294.1713, found 294.1905.

#### 4-(Piperidin-1-yl)-2-(pyridin-2-yl)­quinoline **7a**

^1^H NMR (400 MHz, DMSO-d^6^) δ 8.76–8.72 (1H, m), 8.59 (1H, d, *J* = 7.9 Hz), 8.07 (1H, s), 8.05–7.96 (3H, m), 7.75–7.70 (1H, m), 7.57 (1H, t, *J* = 7.6 Hz), 7.50 (1H, dd, *J* = 7.4, 4.8 Hz), 3.24 (4H, t, *J* = 5.3 Hz), 1.83 (4H, t, *J* = 5.6 Hz), 1.68 (2H, d, *J* = 5.8 Hz). ^13^C NMR (101 MHz, DMSO-d^6^) δ: 158.1, 155.7, 155.5, 149.0, 148.8, 137.2, 129.9, 129.3, 125.6, 124.4, 123.7, 123.0, 121.0, 105.5, 53.1, 25.6, 23.9. ESI/MS calcd for C_19_H_19_N_3_ [M + H]^+^ 290.1657, found 290.1651.

#### 9-(4-Fluorobenzyl)-1-oxa-4,9-diazaspiro­[5.5]­undecan-3-one **8a**

^1^H NMR (500 MHz, DMSO-d^6^) δ 7.93 (1H, s), 7.33–7.30 (2H, m), 7.13 (2H, t, *J* = 8.5 Hz), 3.93 (2H, s), 3.44 (2H, s), 3.07 (2H, s), 2.46 (2H, s), 2.22 (2H, t, *J* = 10.5 Hz), 1.73 (2H, d, *J* = 13.2 Hz), 1.56 (2H, t, *J* = 10.7 Hz). ^19^F NMR (471 MHz, DMSO-d^6^) δ −116.1. ^13^C NMR (100 MHz, DMSO-d^6^) δ: 167.2, 161.2 (d, *J* = 242.3 Hz), 134.7 (d, *J* = 1.9 Hz), 130.5 (d, *J* = 8.0 Hz), 114.8 (d, *J* = 21.1 Hz), 68.1, 62.0, 61.1, 49.0, 48.2, 31.3. HRMS (ES+): *m*/*z* [M + H]^+^ calcd for C_15_H_19_FN_2_O_2_ [M + H]^+^ 279.1509, found 279.1501.

#### 9-(3-Fluorobenzyl)-3,9-diazaspiro­[5.5]­undecane-3-carboxamide **9a**

^1^H NMR (400 MHz, DMSO-d^6^) 7.34 (1H, dd, *J* = 14.0, 7.6 Hz), 7.13–7.03 (3H, m), 5.78 (2H, s), 3.47 (2H, s), 3.24–3.21 (4H, m), 2.33 (4H, bs), 1.45–1.43 (4H, m), 1.33–1.30 (4H, m). ^19^F NMR (471 MHz, DMSO-d^6^) δ −113.9. ^13^C NMR (125 MHz, DMSO-d^6^) δ: 163.4, 161.0, 158.0, 141.9, 129.9 (d, *J* = 8.3 Hz), 124.5, 115.0 (d, *J* = 21 Hz), 113.5 (d, *J* = 21 Hz), 61.6, 48.4, 39.0, 35.0, 29.1. HRMS (ES+): *m*/*z* [M + H]^+^ calcd for C_17_H_24_FN_3_O 306.1976, found 306.2548.

## Supplementary Material



## Notes

Concentration–response curves, synthetic procedures, NMR spectra for the hit compounds **1a**–**9a**, and NMR spectra for key synthetic intermediates (PDF)

## ⊥

DataLoch, Usher Institute, Usher Building, The University of Edinburgh, Edinburgh EH16 4UX, U.K

## #

Assisted Conception Unit, Ninewells Hospital, James Arrott Drive, Dundee DD1 9SY, U.K.

## ∇

Envision Pharma, Savoy Tower, 77 Renfrew Street, Glasgow G2 3BZ, U.K.
