# Supplementary material for: Compounds Reducing Human Sperm Motility as Potential Nonhormonal Contraceptives Identified Using a High-Throughput Phenotypic Screening Platform
Source: ACS Omega. 2025 Nov 27;10(48):59121–34. doi: 10.1021/acsomega.5c08336 (PMC12771125; doi:10.1021/acsomega.5c08336)
Supplement: Supplementary file 1 [file ao5c08336_si_001.pdf]

Compounds reducing human sperm motility as potential non hormonal contraceptives identified using a high-throughput phenotypic screening platform

Anthony Richardson[‡*], Franz S. Gruber[$1], David P. Day[‡], Darren Edwards[‡], Irene Georgiou[‡], Zoe C. Johnston[~], Halimatu Joji[‡], Sarah Martins da Silva[~], Rachel Myles[~2], Neil R. Norcross[‡3], Kevin D. Read[‡], Jason R. Swedlow[§,$], Caroline Wilson[‡], Christopher LR Barratt[~], Ian H. Gilbert[‡]

[~]Reproductive Medicine Research Group, Division of Systems Medicine, School of Medicine, Ninewells Hospital and Medical School, University of Dundee, Dundee, DD19SY, UK

‡ Drug Discovery Unit, Wellcome Centre for Anti-Infectives Research, Division of Biological Chemistry and Drug Discovery, University of Dundee, Dundee, DD1 5EH, UK

[§]University of Dundee, Divisions of Computational Biology and Molecular, Cellular and Developmental Biology, School of Life Sciences, Dundee, UK

[$]National Phenotypic Screening Centre, School of Life Sciences, University of Dundee, Dundee DD1 5EH

*Corresponding author: arichardson001@dundee.ac.uk

[1] Current address for Franz S. Gruber is DataLoch, Usher Institute, Usher Building, The University of Edinburgh, EH16 4UX

[2] Current address for Rachel Myles is Assisted Conception Unit, Ninewells Hospital, James Arrott Drive, Dundee, United Kingdom, DD1 9SY

[3] Current address for Neil R. Norcross is Envision Pharma, Savoy Tower, 77 Renfrew Street, Glasgow, G2 3BZ, UK

Supporting Information

Contents

Concentration-response curves for compounds **1a – 9a** in sperm motility assay and HepG2 viability assay

General experimental procedures to access hit compounds **1a – 9a**

^{1}H NMR, ^{19}F NMR and ^{13}C NMR spectra for hit compounds **1a – 9a**

^{1}H NMR of intermediates **1o**, **2g**, **2h**, **5l**, **5m**

Sperm motility concentration-response curves

Figure S1 shows the concentration-response curves for the primary hits in the high-throughput sperm motility assay at 3 different incubation times (10 minutes, 3 hours and 6 hours).

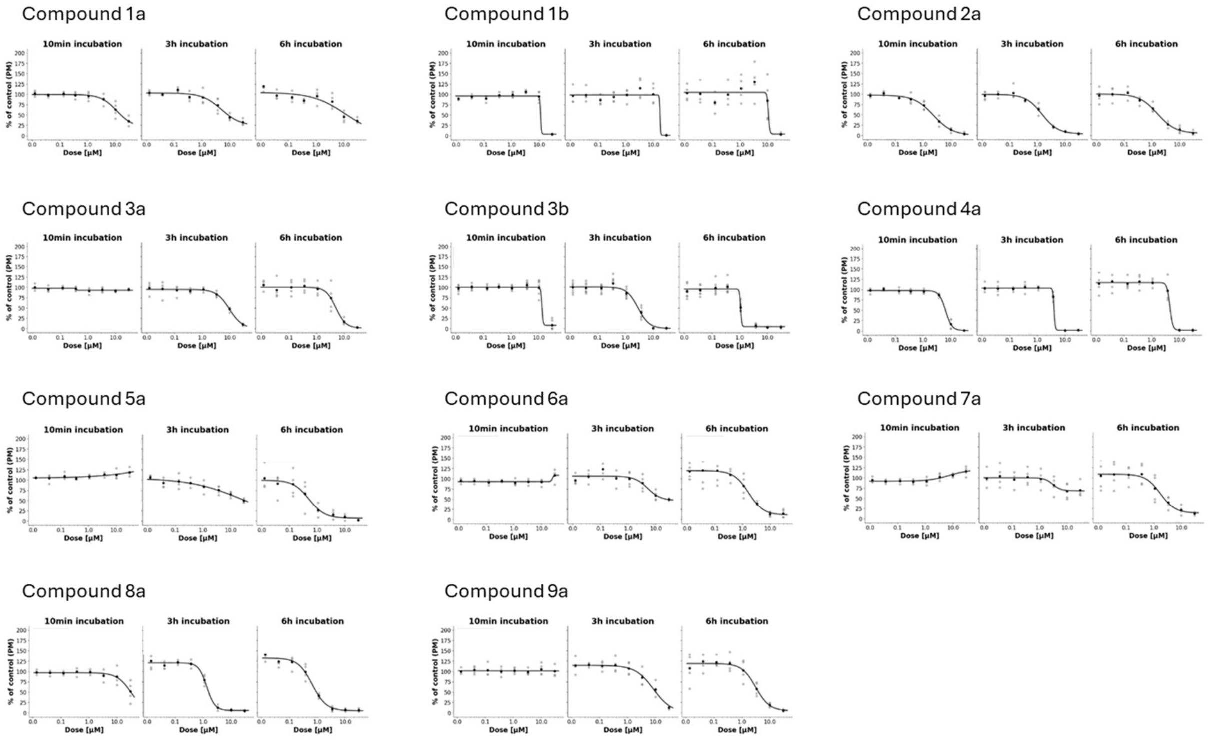

Figure S1: Concentration-response curves from high throughput sperm motility assay. The x-axis is concentration of compound in µM and the y-axis is percentage of progressively motile cells relative to DMSO control. The experiments were carried out with a minimum of 2 biological replicates (i.e. minimum of 2 different donor pools) and each biological replicate was run with 2 technical replicates. The individual data points are represented by grey dots and the average values across all replicates are represented by black dots.

HepG2 concentration-response curves

Figure S2 shows the concentration-response curves for the primary hits in the high-throughput HepG2 viability assay (*note: compounds 3a and 6a were not tested in this assay*).

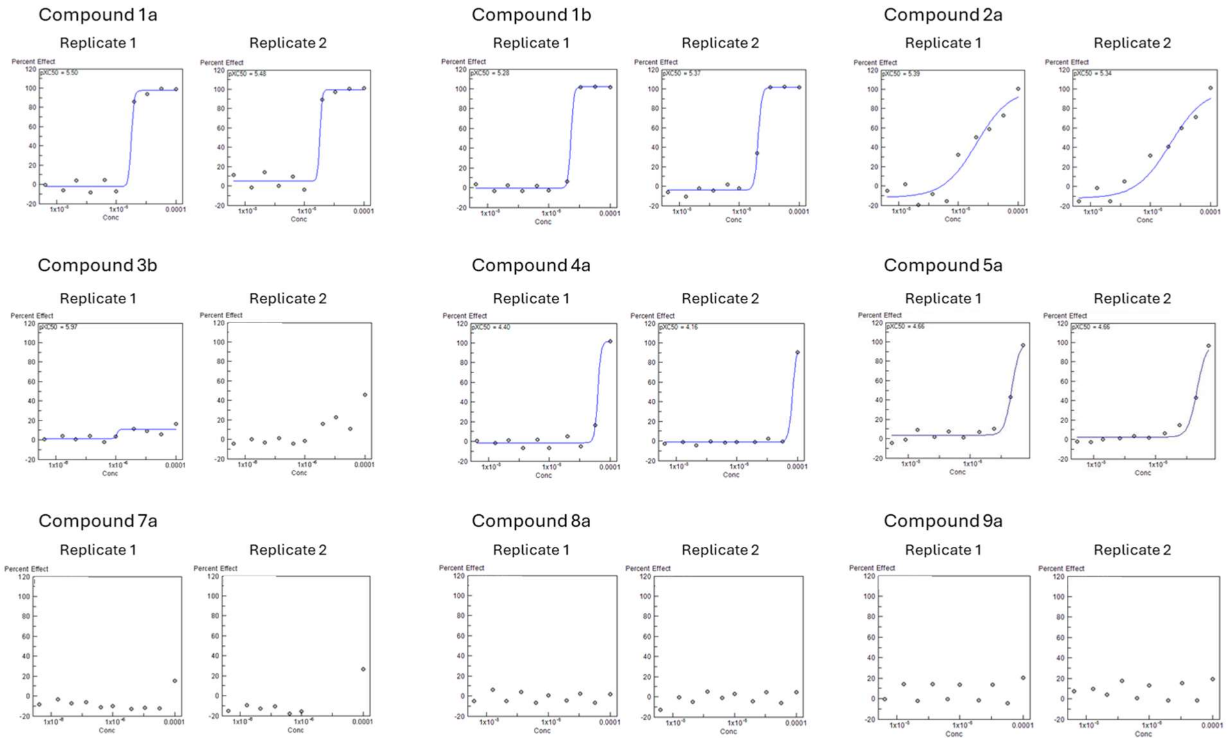

Figure S2: Concentration-response curves from high throughput HepG2 viability assay. Each compound was tested in duplicate and the curves of each replicate are shown. The reported values in the tables are an average of the 2 pEC_{50} values obtained. The x-axis shows concentration in M and the y-axis shows % effect, relative to positive and negative controls (i.e. negative control used to determine 0% effect, positive control used to determine 100% effect).

Synthetic routes

Hit compounds **1a**, **6b** and **7a** were repurchased from commercial suppliers including enamine and Sigma Aldrich. For the remaining hit compounds **1b**, **2a**, **3a**, **3b**, **4a**, **5a**, **8a** and **9a**, their synthetic routes are described below.

1a analytical report

^{1}H NMR (400 MHz, DMSO-d^6) δ 11.53 (1H, bs), 8.82 (1H, d, J = 9.2 Hz), 8.45 (1H, d, J = 2.6 Hz), 8.30 (1H, dd, J = 9.2, 2.7 Hz), 7.97 (1H, d, J = 2.8 Hz), 7.53 (1H, dd, J = 8.8, 2.8 Hz), 7.09 (1H, d, J = 8.8 Hz), 5.75 (1H, s). ^{13}C NMR (101 MHz, DMSO-d^6) δ 162.6, 155.3, 142.6, 141.2, 133.9, 130.0, 124.7, 123.8, 123.6, 122.4, 120.8, 119.4, 119.2.

6b analytical report

^{1}H NMR (400 MHz, DMSO) δ 10.32 (1H, s), 7.17 – 7.09 (5H, m), 5.95 (1H, s), 2.51 (6H, s), 2.26 (3H, s), 2.18 (3H, s). ^{13}C NMR (100 MHz, DMSO-d^6) δ 168.1, 156.8, 150.4, 145.8, 138.4, 130.2, 129.8, 117.3, 116.3, 90.0, 23.4, 20.2, 14.0. HRMS (ES+): m/z [M + H]+ calcd for $C_{17}H_{19}N_5$ [M+H]+ 294.1713, found 294.1905.

7a analytical report

^{1}H NMR (400 MHz, DMSO-d^6) δ 8.76 – 8.72 (1H, m), 8.59 (1H, d, J = 7.9 Hz), 8.07 (1H, s), 8.05 – 7.96 (3H, m), 7.75 – 7.70 (1H, m), 7.57 (1H, t, J = 7.6 Hz), 7.50 (1H, dd, J = 7.4, 4.8 Hz), 3.24 (4H, t, J = 5.3

Hz), 1.83 (4H, t, J = 5.6 Hz), 1.68 (2H, d, J = 5.8 Hz). ^{13}C NMR (101 MHz, DMSO-d^6) δ 158.1, 155.7, 155.5, 149.0, 148.8, 137.2, 129.9, 129.3, 125.6, 124.4, 123.7, 123.0, 121.0, 105.5, 53.1, 25.6, 23.9. ESI/MS calcd for $C_{19}H_{19}N_3$ [M+H]$^+$ 290.1657, found 290.1651.

1b synthetic route

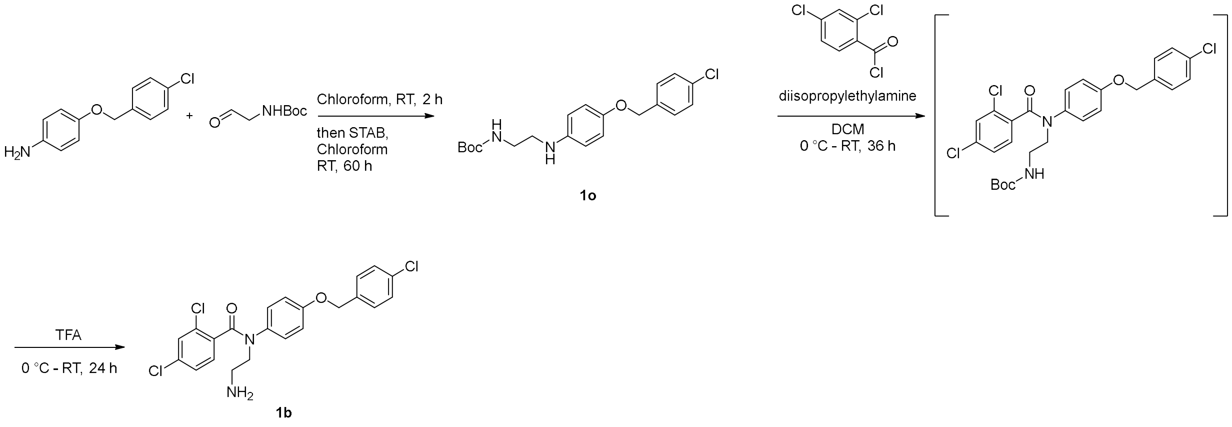

4-[(4-Chlorophenyl)methoxy]aniline (350 mg) was dissolved in chloroform (5 mL), N-Boc-2-aminoacetaldehyde (238 mg) in chloroform (3 mL) was added and the mixture was stirred at room temperature (RT) for 2 hours. Subsequently, sodium triacetoxyborohydride (413 mg) was added and the reaction stirred at RT for 60 hours. Water (3 mL) was added and the organic layer was separated by filtration through a phase separator then directly purified by column chromatography (0-10% methanol/DCM) and the product fractions concentrated to dryness. The solid obtained was further purified by trituration in heptane/diethyl ether (1:1) to afford tert-butyl (2-((4-((4-chlorobenzyl)oxy)phenyl)amino)ethyl)carbamate (**1o**) as an off-white solid (293 mg). 1H NMR (500 MHz, $CDCl_3$) 7.35 (4H, s), 7.33 (1H, s), 7.11 (2H, d, J = 8.9 Hz), 6.94 (2H, d, J = 9.0 Hz), 5.01 (2H, s), 2.07 (2H, s), 1.50-1.43 (12H, m). LCMS (ES+) m/z 377 [M + H]$^+$.

1o (293 mg) was dissolved in DCM (10 mL), and N,N-diisopropylethylamine (210 µL) added and the mixture cooled to 0 °C. 2,4-dichlorobenzoyl chloride (209 mg) was added portion-wise over 1 minute, and then the mixture was stirred at 0 °C for 5 minutes and allowed to warm to RT and stirred for 36 hours. The mixture was then cooled to 0 °C, to which trifluoroacetic acid (1.2 mL) was added and the mixture allowed to warm to RT and stirred for 24 hours. The reaction mixture was concentrated under reduced pressure and purified by SCX column (base capture), followed by column chromatography (0-10% methanol/DCM) to afford N-(2-aminoethyl)-2,4-dichloro-N-[4-[(4-chlorophenyl)methoxy]phenyl]benzamide (**1b**) (100 mg), 13% yield (over 3 steps) as a colourless oil.

^{1}H NMR of 1b (400 MHz, $CDCl_3$) δ 7.37-7.29 (4H, m), 7.23 (1H, d, *J* = 1.5 Hz), 7.07-7.02 (4H, m), 6.76 (2H, d, *J* = 8.9 Hz), 4.93 (2H, s), 3.95 (2H, t, *J* = 6.2 Hz), 2.94 (2H, t, *J* = 6.5 Hz), 1.40 (2H, bs).

^{13}C NMR of 1b (101 MHz, $CDCl_3$) δ 167.9, 157.8, 135.6, 135.0, 134.9, 134.8, 134.2, 131.5, 129.51, 129.45, 129.1, 129.0, 128.9, 126.8, 115.5, 69.5, 52.5, 40.1.

HRMS (ES+): m/z [M + H]+ calcd for $C_{22}H_{19}Cl_3N_2O_2$ [M+H]+ 449.0585, found 449.0573.

2a synthetic route

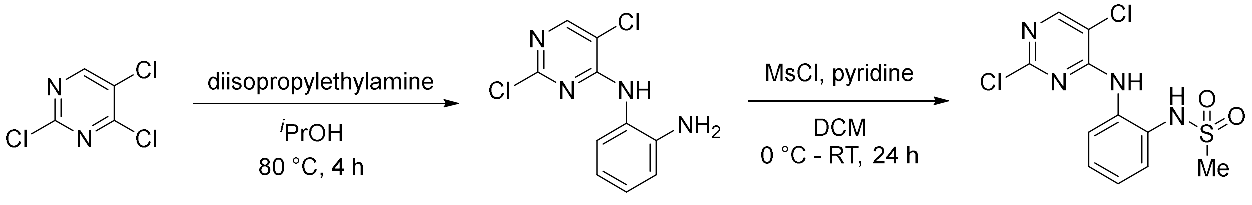

2g **2h**

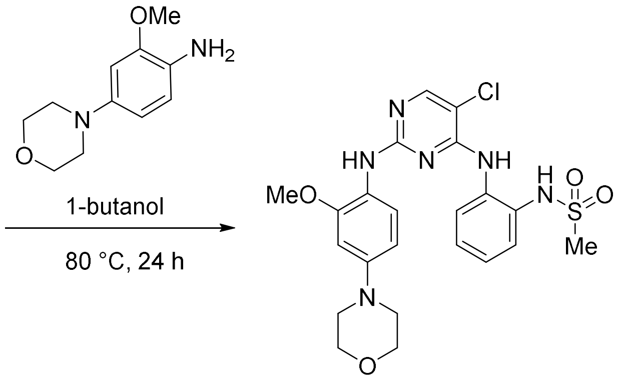

2a

A solution of 2,4,5-trichloro-pyrimidine (2 g), N,N-diisopropylethylamine (2.97 mL) and 1,2 phenylenediamine (1.03 mL) in isopropanol (20 mL) was heated at 80 °C. Upon completion, the reaction was allowed to cool to RT. The precipitated solid was filtered, dissolved in water, adjusted to pH 7 using 1M HCl, and the suspension left to stir for 30 min at RT. Filtration afforded N1-(2,5-dichloropyrimidin-4-yl)benzene-1,2-diamine (**2g**) as a white solid (2.13 g, 75% yield). ^{1}H NMR (500 MHz, DMSO) δ 8.97 (s, 1H), 8.25 (s, 1H), 7.05 – 6.97 (m, 2H), 6.76 (dd, J = 8.0, 1.4 Hz, 1H), 6.57 (td, J = 7.5, 1.4 Hz, 1H), 4.93 (s, 2H). HRMS (ES+): m/z [M + H]+ calcd for $C_{10}H_8Cl_2N_4$ $[M+H]^+$ 255.0199, found 255.0212.

2g (1.00 g) was dissolved in DCM (30 mL), cooled to 0 °C and pyridine (950 µL, 11.8 mmol) was added dropwise. Subsequent dropwise addition of methanesulfonyl chloride (330 µL) at 0 °C, followed by allowing to attain RT and was stirred for 24 h. The reaction was poured into H_2O, and the organic solvent was evaporated off. The pH of the resulting suspension was adjusted to 7 using aq. 1M HCl solution. The white solid was filtered off to afford N-(2-((2,5-dichloropyrimidin-4-yl)amino)phenyl)methanesulfonamide (**2h**) (1.22 g, 88% yield). ^{1}H NMR (500 MHz, DMSO) δ 9.26 (s, 1H), 9.06 (s, 1H), 8.39 (s, 1H), 7.64 – 7.59 (m, 1H), 7.50 – 7.45 (m, 1H), 7.34 – 7.28 (m, 2H), 2.99 (s, 3H). HRMS (ES+): m/z [M + H]+ calcd for $C_{11}H_{10}Cl_2N_4O_2S$ $[M+H]^+$ 332.9974, found 332.9974.

2h (150 mg mmol) and 2-methoxy-4-morpholinoaniline (103 mg) were dissolved in 1-butanol (2.25 mL), to which 4M HCl in dioxane (135 µL) was added and the reaction was heated at 80 °C for 24 hours. The resulting precipitate was filtrated and washed several times with a minimal volume of diethyl ether. This solid was dissolved in DCM (5 mL), 10% aq. K_2CO_3 (5 mL) was added and the mixture stirred for 15 minutes. The organic layer was separated, and residual solvents removed under reduced pressure. The residue was purified by column chromatography on silica gel using A (DCM) and B (10%

MeOH–NH_3 in DCM), followed by a second purification using column chromatography on silica gel using A (heptane) and B (ethyl acetate, 20-60%) to afford the desired product N-(2-((5-chloro-2-((2-methoxy-4-morpholinophenyl)amino)pyrimidin-4-yl)amino)phenyl)methanesulfonamide (**2a**) as a cream solid (21 mg, 9%).

^{1}H NMR of 2a (500 MHz, $CDCl_3$) δ 8.08 (s, 1H), 7.76 (1H, d, *J* = 8.8 Hz), 7.69 – 7.65 (1H, m), 7.55 – 7.50 (1H, m), 7.39 (1H, s), 7.35 – 7.29 (2H, m), 7.25 (1H, s), 6.94 (1H, s), 6.47 (1H, d, *J* = 2.5 Hz), 6.31 (1H, dd, *J* = 8.8, 2.6 Hz), 3.88 – 3.84 (4H, m), 3.83 (3H, s), 3.11 – 3.06 (4H, m), 2.90 (3H, s).

^{13}C NMR of 2a (125 MHz, $CDCl_3$) δ 158.1, 156.7, 155.3, 149.6, 147.7, 133.0, 130.7, 127.6, 127.0, 126.5, 125.9, 122.1, 120.6, 107.8, 104.8, 100.2, 67.1, 55.8, 50.5, 39.8.

HRMS of 2a (ES+): m/z [M + H]+ calcd for $C_{22}H_{25}ClN_6O_4S$ [M+H]+ 505.1419, found 505.1425.

__3a synthetic route__

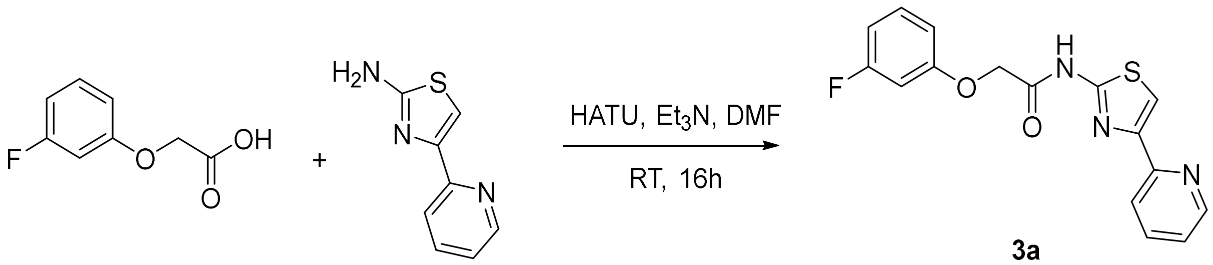

3a

To a microwave vial was added 2-amino-4-(2-pyridyl)thiazole (178 mg), (3-fluoro-phenoxy)-acetic acid (171 mg), HATU (382 mg) and triethylamine (102 mg), to which DMF (1.5mL) was added and the reaction stirred at rt for 16 h. The reaction was evaporated under reduced pressure, and the green residue dissolved in MeOH-Water (9:1, 5 mL). This mixture was purified by high pH reverse phase preparative HPLC (5–95% gradient, 0.1% NH_3 in water / MeCN), and after evaporation of organic solvents the desired product was a white solid, 2-(3-fluorophenoxy)-N-[4-(2-pyridyl)thiazol-2-yl]acetamide (**3a**) (205 mg, 59% yield).

^{1}H NMR of 3a ($CDCl_3$, 400 MHz) 9.76 (1H, bs), 8.66-8.64 (1H, m), 7.92 (1H, d, J = 7.9 Hz), 7.75 (1H, td, J = 7.7, 1.8 Hz), 7.72 (1H, s), 7.32 (1H, td, J = 8.3, 6.7 Hz), 7.23 (1H, ddd, J = 7.5, 4.8, 1.1 Hz), 6.82-6.78 (2H, m), 6.75 (1H, dt, J = 10.2, 2.4 Hz), 4.73 (2H, s).

^{19}F NMR of 3a (471 MHz, DMSO-d^6) δ -110.07 - -110.13 (m).

^{13}C NMR of 3a ($CDCl_3$, 100 MHz) 166.8, 163.8 (d, J = 247.4 Hz), 158.0 (d, J = 10.8 Hz), 156.6, 152.4, 150.3, 149.9, 137.0, 131.1 (d, J = 9.8 Hz), 123.0, 120.8, 112.5, 110.5 (d, J = 2.9 Hz), 109.9 (d, J = 21.2 Hz), 103.2 (d, J = 25.4 Hz), 67.2.

HRMS of 3a (ES+): m/z [M + H]+ calcd for $C_{16}H_{12}FN_3O_2S$ [M+H]+ 330.0713, found 330.0711.

3b synthetic route

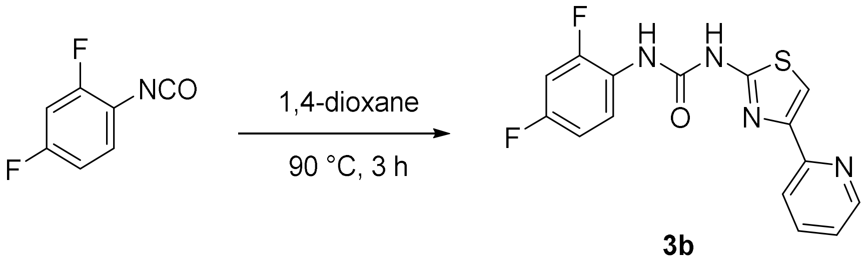

3b

A mixture of 2-amino-4-(2-pyridyl)thiazole (200 mg) and 1,4-dioxane (5 mL) was prepared at RT, to which 2,4-difluoro-1-isocyanato-benzene (134 µL) was added to the mixture. The reaction was heated to 90 °C in a sealed tube for 3 hours. A beige precipitate had formed, the mixture was cooled, ethyl acetate (10 mL) added, and the resultant solid was filtered off, washing with diethyl ether (3 x 5 mL). Successive washings of the solid with ethyl acetate (10 mL), followed by diethyl ether (2 x 10 mL), suspending the solid in acetonitrile (15 mL) and vacuum drying yielded 2-(3-fluorophenoxy)-N-(4-(pyridin-2-yl) thiazol-2-yl)acetamide (**3b**) as a cream solid (375 mg, 59%).

^{1}H NMR of 3b (400 MHz, DMSO-d^6) δ 10.97 (1H, s), 8.92 (1H, bs), 8.60 (1H, d, *J* = 4.5 Hz), 8.10-8.06 (1H, m), 7.95 (1H, d, *J* = 7.7 Hz), 7.89 (1H, t, *J* = 7.8 Hz), 7.77 (1H, s), 7.35 (2H, td, *J* = 11.8. 5.5 Hz), 7.10 (1H, t, *J* = 8.0 Hz).

^{19}F NMR of 3b (471 MHz, DMSO-d^6) δ -116.6 - -116.1 (m), -124.1.

^{13}C NMR of 3b (101 MHz, DMSO-d^6) δ 159.3 (s), 158.7 (d, *J* = 11.8 Hz), 156.3 (d, *J* = 11.5 Hz), 153.8 (d, *J* = 12.4 Hz), 151.4 (dd, *J* = 14.6, 9.8 Hz), 149.0 (s), 148.5 (s), 137.7 (s), 123.0 (d, *J* = 3.4 Hz), 122.9 (s), 122.5 (d, *J* = 9.1 Hz), 120.2 (s), 111.4 (d, *J* = 3.4 Hz), 111.1 (d, *J* = 3.2 Hz), 103.9 (dd, *J* = 26.9, 23.6 Hz).

HRMS of 3b (ES+): m/z [M + H]+ calcd for $C_{15}H_{10}F_2N_4OS$ $[M+H]^+$ 333.0622, found 333.0618.

4a synthetic route

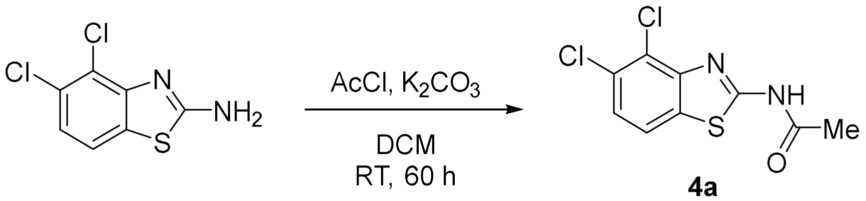

4a

To a microwave vial was added 4,5-dichloro-1,3-benzothiazol-2-amine (302 mg) dissolved in DCM (8 mL), followed by the addition of potassium carbonate (248 mg) and acetyl chloride (118 µL). The mixture was stirred at rt for 65 h. The reaction mixture was diluted with DCM (5 mL) and water (5 mL), the organic layer separated, and solvents were removed under reduced pressure. The resulting residue was purified by column chromatography (0-10% methanol/DCM) to afford the desired product N-(4,5-dichlorobenzo[d]thiazol-2-yl)acetamide (**4a**) as an off-white solid (215 mg, 57%).

^{1}H NMR of 4a (400 MHz, DMSO-d^6) δ 9.84 (1H, s), 7.95 (1H, d, *J* = 8.8 Hz), 7.78 (1H, d, *J* = 8.8 Hz), 2.15 (3H, s).

^{13}C NMR of 4a (101 MHz, DMSO-d^6) δ 169.0, 138.4, 132.8, 130.8, 125.9, 124.6, 120.8, 110.2, 23.5.

HRMS of 4a (ES+): m/z [M + H]+ calcd for $C_9H_6Cl_2N_2OS$ [M+H]+ 260.9651, found 260.9656.

5a synthetic route

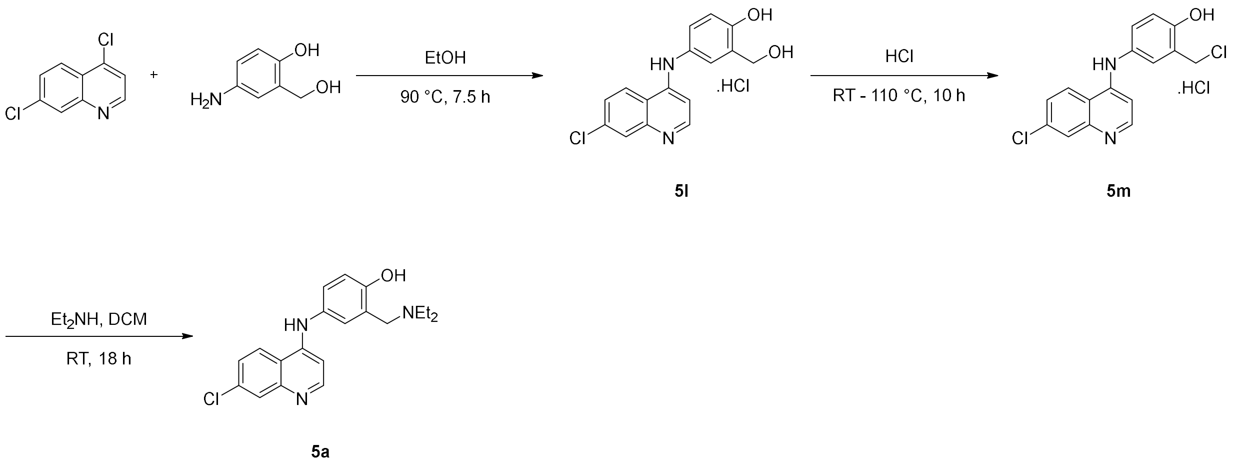

4-Amino-2-(hydroxymethyl)phenol (1.00 g) was dissolved in ethanol (25mL), to which 4,7-dichloro quinoline (1.42 g) was added and the mixture heated to 90 °C for 7.5 hours. The reaction was then cooled to RT. Upon precipitation, the solid was filtered, washed with ethanol (2 x 20 mL) and dried under reduced pressure to yield the desired product 4-[(7-chloro-4-quinolyl)amino]-2-(hydroxymethyl)phenol hydrochloride **5l** (2.12 g, 79% yield) as a green solid. ^{1}H NMR (500 MHz, DMSO-d^6) δ 14.12 (1H, bs), 10.86 (1H, s), 9.83 (1H, s), 8.74 (1H, d, J = 9.1 Hz), 8.46 (1H, d, J = 7.0 Hz), 8.06 (1H, d, J = 2.0 Hz), 7.85 (1H, dd, J = 9.1, 2.0 Hz), 7.34 (1H, d, J = 2.4 Hz), 7.13 (1H, dd, J = 8.4, 2.6 Hz), 6.96 (1H, d, J = 8.4 Hz), 6.66 (1H, d, J = 7.0 Hz), 5.13 (1H, bs), 4.54 (2H, s). LCMS (ES+) m/z 301 [M + H]$^+$.

4-[(7-Chloro-4-quinolyl)amino]-2-(hydroxymethyl)phenol hydrochloride (2.12 g) was dissolved in concentrated HCl (80 mL) and heated to 110 °C for 10 hours. Upon cooling, residual acid was removed, and the resulting solid was triturated with a minimal volume of diethyl ether and filtered, washed with a minimal volume of diethyl ether and dried under reduced pressure to afford the desired product 2-(chloromethyl)-4-[(7-chloro-4-quinolyl)amino]phenol hydrochloride **5m** (1.61 g, 68% yield) as a green solid. ^{1}H NMR (500 MHz, DMSO-d^6) δ 14.21 (1H, bs), 10.88 (1H, s), 10.38 (1H, s), 8.72 (1H, d, J = 9.1 Hz), 8.50 (1H, d, J = 7.0 Hz), 8.06 (1H, d, J = 1.9 Hz), 7.87 (1H, dd, J = 9.1, 1.9 Hz), 7.42 (1H, d, J = 2.5 Hz), 7.28 (1H, dd, J = 8.6, 2.6 Hz), 7.06 (1H, d, J = 8.6 Hz), 6.65 (1H, d, J = 8.6 Hz), 4.75 (2H, s). LCMS (ES+) m/z 315 [M + H]$^+$ for methanol substituted product.

2-(Chloromethyl)-4-[(7-chloro-4-quinolyl)amino]phenol hydrochloride (200mg) was dissolved in DCM (6 mL), to which diethyl amine (230 µL) was added and the reaction stirred for 18 h at rt. The mixture was then diluted with DCM, washed with water, organics separated and concentrated under reduced pressure. The crude solid was purified by column chromatography (0-10% methanol/DCM) to afford the desired product 4-[(7-chloro-4-quinolyl)amino]-2-(diethylaminomethyl)phenol (**5a**) (126 mg, 59% yield) as a pale yellow solid.

^{1}H NMR of 5a (400 MHz, DMSO-d^6) δ 8.85 (1H, s), 8.40 (1H, d, *J* = 9.1 Hz), 8.36 (1H, d, *J* = 5.4 Hz), 7.84 (1H, d, *J* = 2.2 Hz), 7.51 (1H, dd, *J* = 9.0, 2.2 Hz), 7.08-7.06 (2H, m), 6.78 (1H, d, *J* = 8.2 Hz), 6.56 (1H, d, *J* = 5.4 Hz), 3.74 (2H, s), 2.58 (4H, q, *J* = 7.1 Hz), 1.04 (6H, t, *J* = 7.1 Hz).*OH/NH signal not observed.

^{13}C NMR of 5a (101 MHz, DMSO-d^6) δ 161.7, 155.0, 151.8, 149.5, 133.6, 130.4, 127.5, 125.3, 124.6, 124.5, 124.2, 124.0, 117.7, 116.0, 100.5, 54.8, 45.9, 11.1.

HRMS of 5a (ES+): m/z [M + H]+ calcd for $C_{20}H_{22}ClN_3O$ [M+H]+ 356.1530, found 356.1547.

8a synthetic route

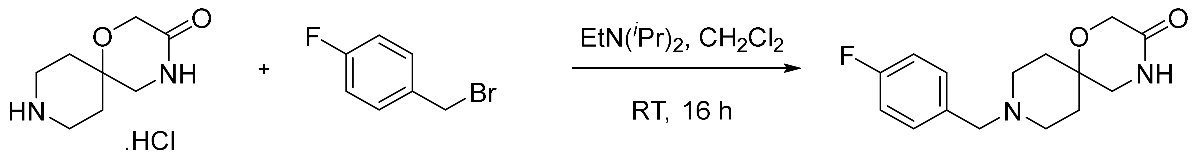

8a

To a round bottom flask was added 1-Oxa-4,9-diazaspiro[5.5]undecan-3-one hydrochloride (50 mg) and DCM (5 mL) at rt, to which N,N-diisopropylethylamine (150 µL) was added, followed by the dropwise addition (over 1 min) of 1-(bromomethyl)-4-fluorobenzene (36 µL) and the mixture stirred for 18 hours. The reaction mixture was evaporated under reduced pressure, dissolved in methanol (2 mL), and purified via SCX-2g cartridge, eluting with MeOH-NH_3 (7M, 3 mL) and methanol (5 mL). 9-[(4-fluorophenyl)methyl]-1-oxa-4,9-diazaspiro[5.5]undecan-3-one (**8a**) (4.5 mg, 7% yield) was isolated as a white solid.

^{1}H NMR of 8a (500 MHz, DMSO-d^6) δ 7.93 (1H, s), 7.33-7.30 (2H, m), 7.13 (2H, t, *J* = 8.5 Hz), 3.93 (2H, s), 3.44 (2H, s), 3.07 (2H, s), 2.46 (2H, s), 2.22 (2H, t, *J* = 10.5 Hz), 1.73 (2H, d, *J* = 13.2 Hz), 1.56 (2H, t, *J* = 10.7 Hz).

^{19}F NMR of 8a (471 MHz, DMSO-d^6) δ -116.1.

^{13}C NMR of 8a (100 MHz, DMSO-d^6) δ 167.2, 161.2 (d, J = 242.3 Hz), 134.7 (d, J = 1.9 Hz), 130.5 (d, J = 8.0 Hz), 114.8 (d, J = 21.1 Hz), 68.1, 62.0, 61.1, 49.0, 48.2, 31.3.

HRMS of 8a (ES+): m/z [M + H]+ calcd for $C_{15}H_{19}FN_2O_2$ $[M+H]^+$ 279.1509, found 279.1501.

9a synthetic route

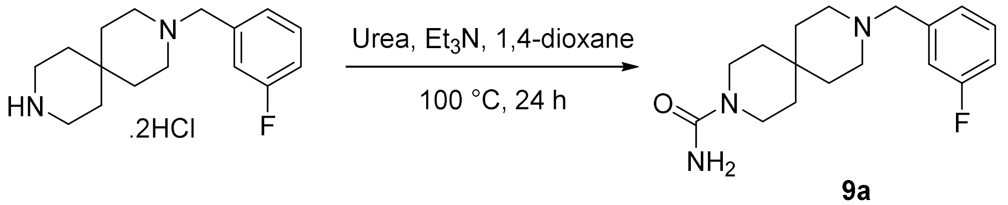

9a

A mixture of 3-[(3-fluorophenyl)methyl]-3,9-diazaspiro[5.5]undecane dihydrochloride (80 mg), urea (72 mg) and triethylamine (100 µL) in 1,4-dioxane (4mL) was heated at 100 °C for 24 hours. The reaction mixture was cooled, solvents evaporated in vacuo, dissolved in a minimal volume of methanol and absorbed onto silica gel. The desired product was eluted on silica gel chromatography using 0-100% 20% MeOH-NH_3/DCM. Evaporation of remaining organic solvents yielded the desired product 9-(3-fluorobenzyl)-3,9-diazaspiro[5.5]undecane-3-carboxamide (**9a**) as a white solid (45 mg, 59% yield).

^{1}H NMR of 9a (400 MHz, DMSO-d^6) 7.34 (1H, dd, *J* = 14.0, 7.6 Hz), 7.13-7.03 (3H, m), 5.78 (2H, s), 3.47 (2H, s), 3.24-3.21 (4H, m), 2.33 (4H, bs), 1.45-1.43 (4H, m), 1.33-1.30 (4H, m).

^{19}F NMR of 9a (471 MHz, DMSO-d^6) δ -113.9.

^{13}C NMR of 9a (125 MHz, DMSO-d^6) δ 163.4, 161.0, 158.0, 141.9, 129.9 (d, J = 8.3 Hz), 124.5, 115.0 (d, J = 21 Hz), 113.5 (d, J = 21 Hz), 61.6, 48.4, 39.0, 35.0, 29.1.

HRMS of 9a (ES+): m/z [M + H]$^+$ calcd for $C_{17}H_{24}FN_3O$ 306.1976, found 306.2548.

NMR data of final compounds

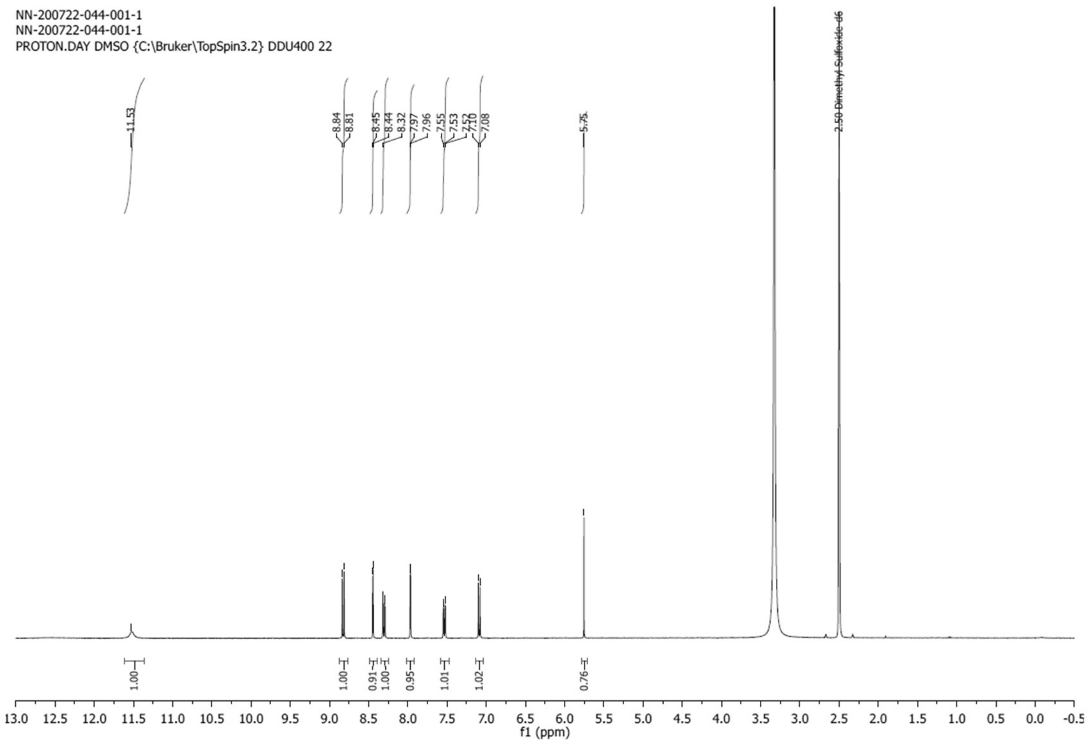

Figure S3: ^{1}H NMR (400 MHz, DMSO-d^6) of **1a**

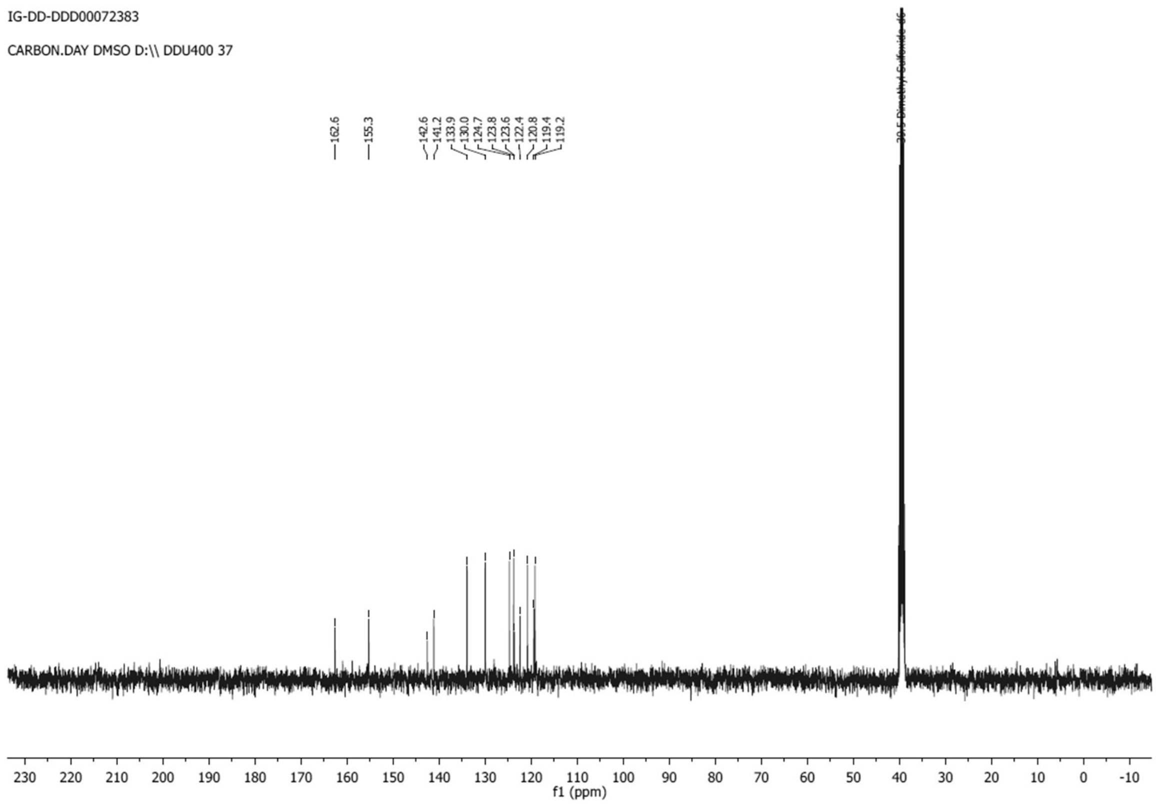

Figure S4: ^{13}C NMR (101 MHz, DMSO-d^{6}) of **1a**

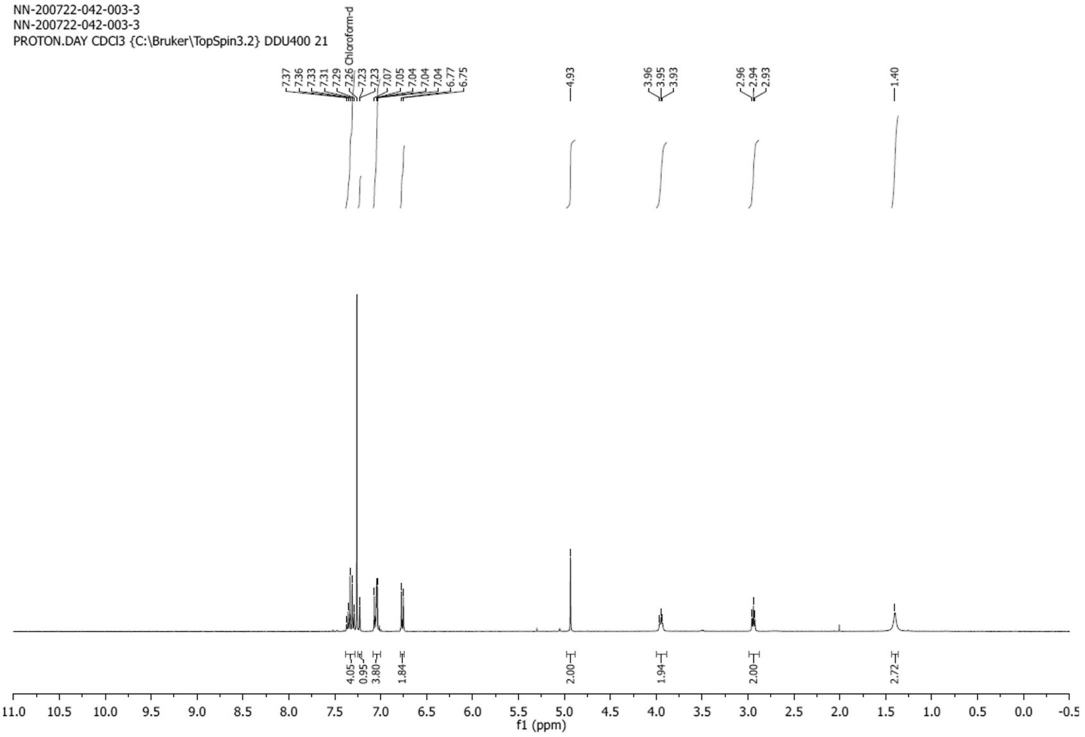

Figure S5: ^{1}H NMR (400 MHz, $CDCl_3$) of **1b**

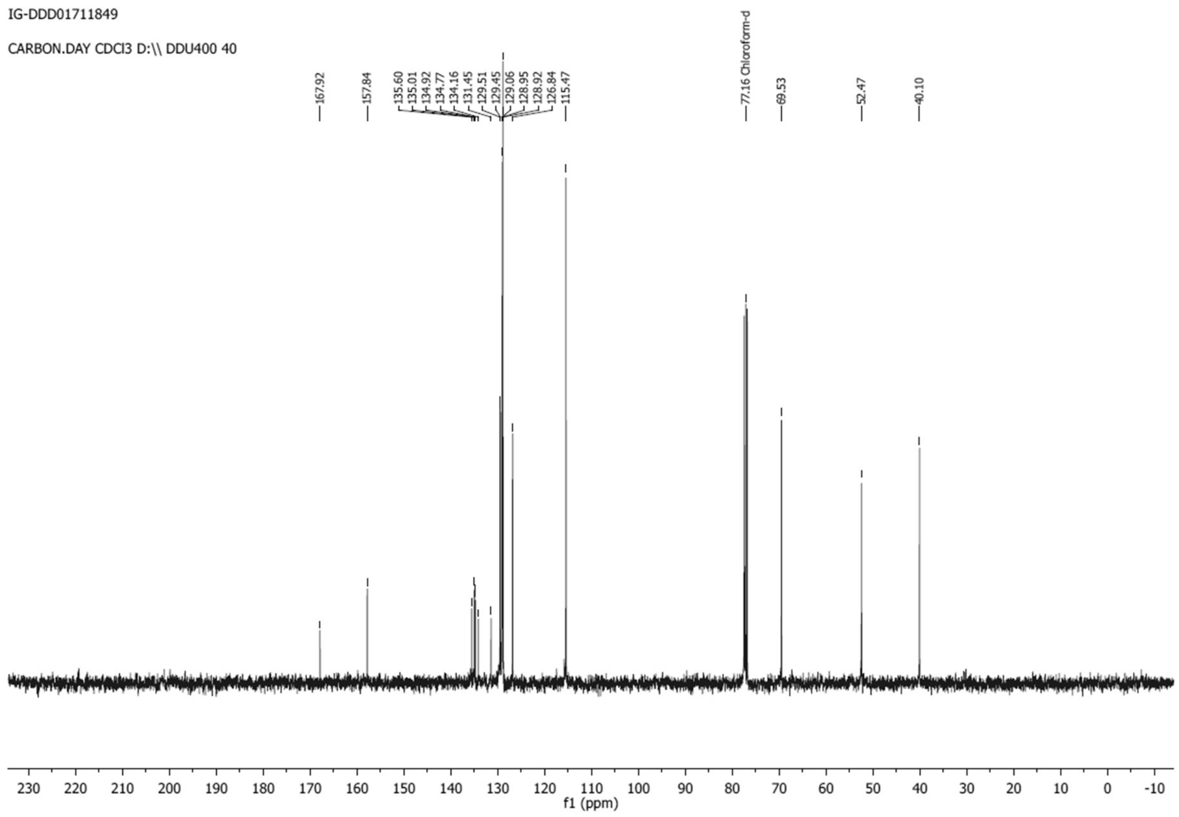

Figure S6: ^{13}C NMR (100 MHz, $CDCl_3$) of **1b**

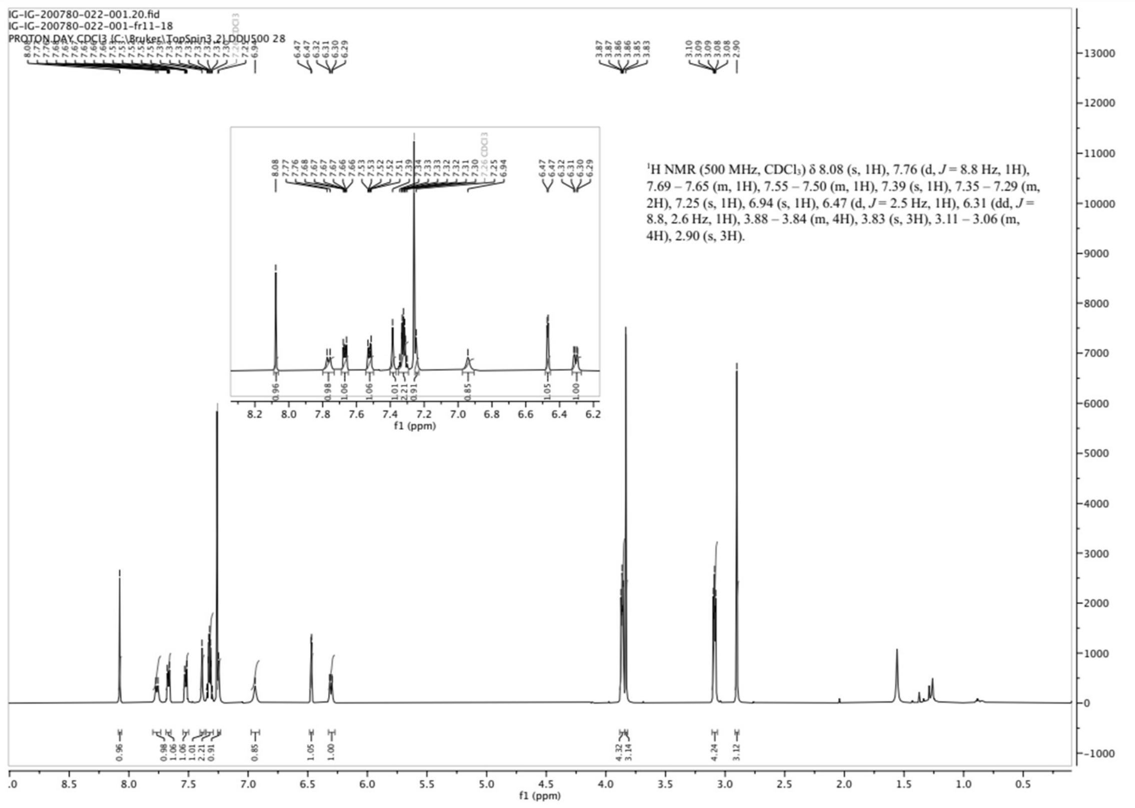

Figure S7: ^{1}H NMR (400 MHz, $CDCl_3$) of **2a**

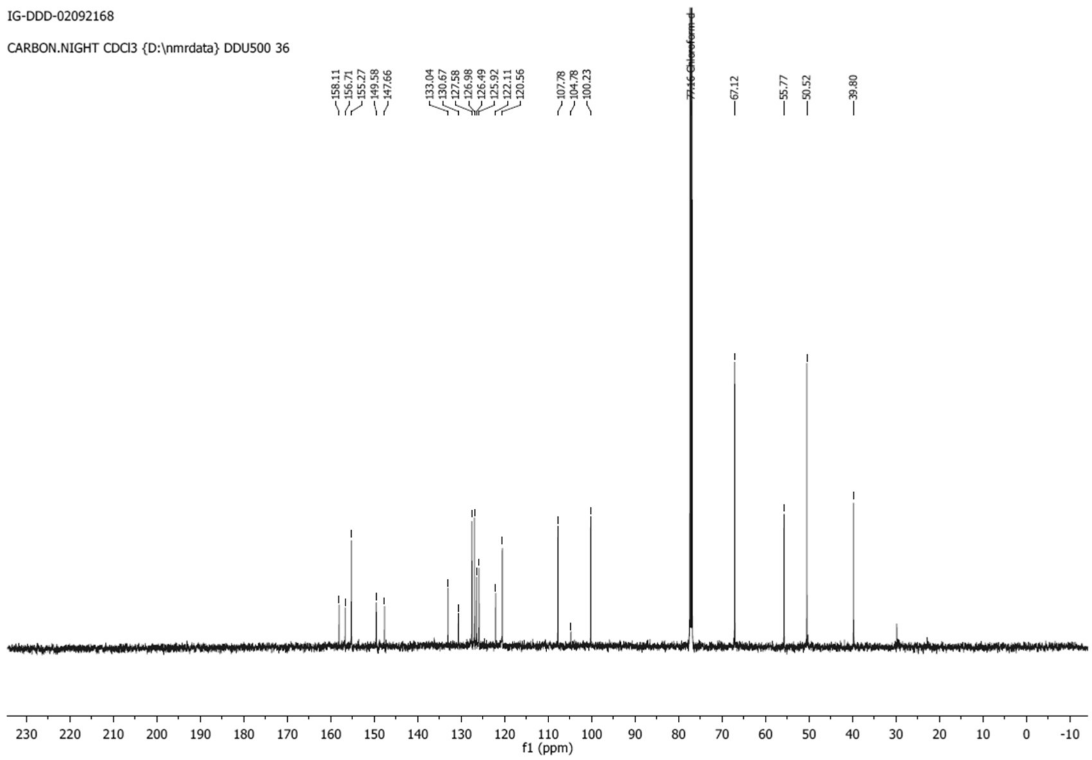

Figure S8: ^{13}C NMR (125 MHz, $CDCl_3$) of **2a**

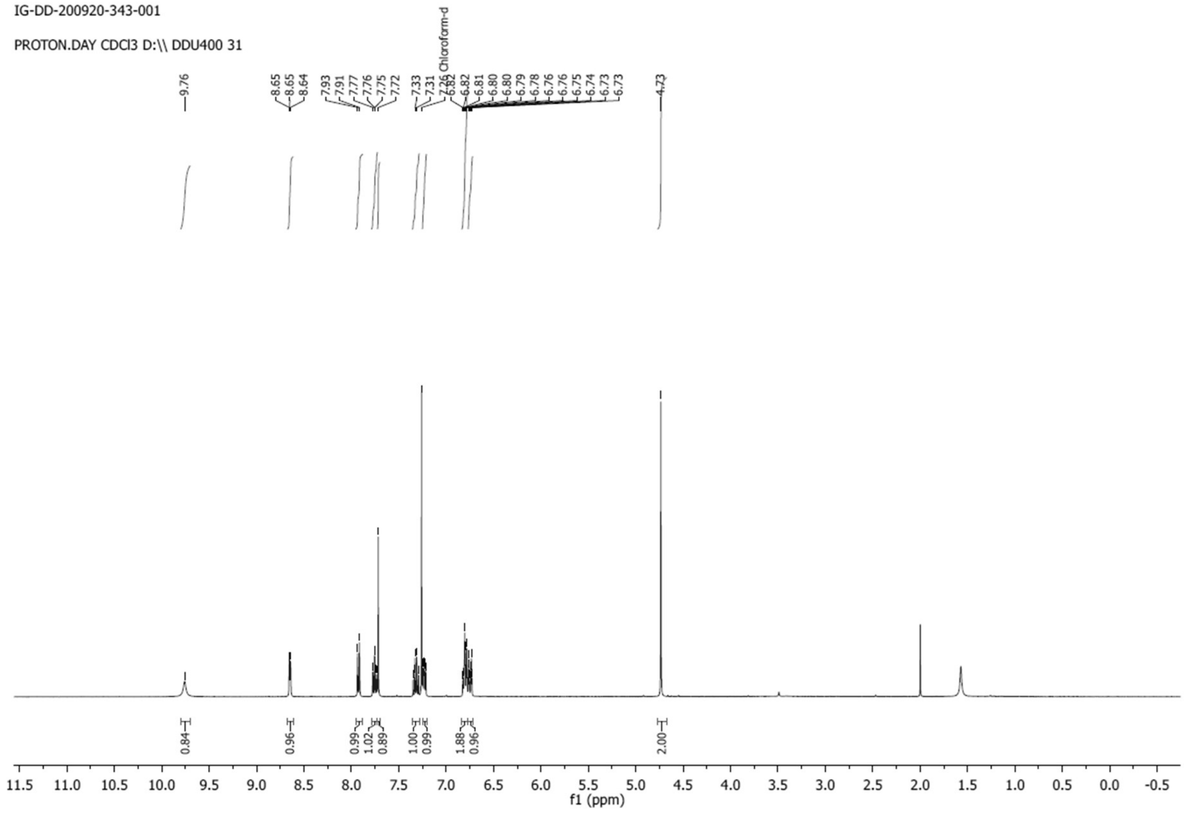

Figure S9: ^{1}H NMR (400 MHz, $CDCl_3$) of **3a**

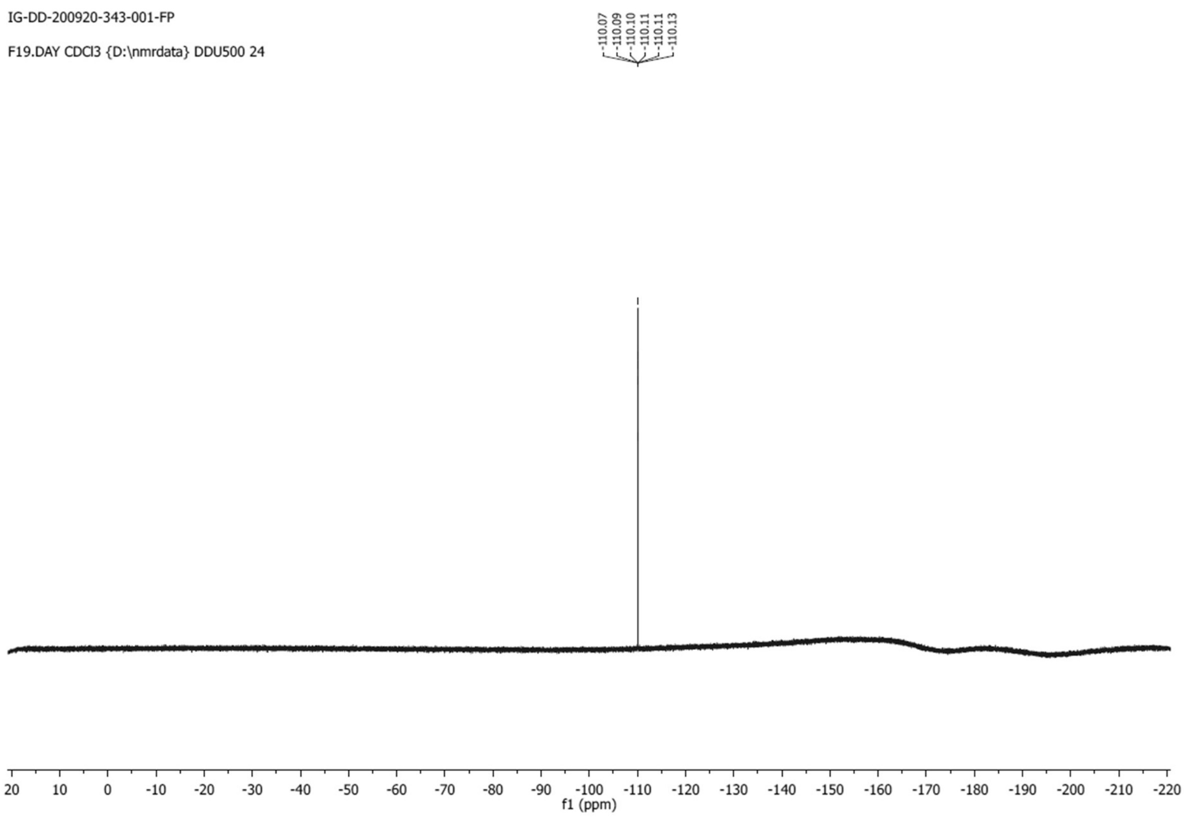

Figure S10: ^{19}F NMR (471 MHz, $CDCl_3$) of **3a**

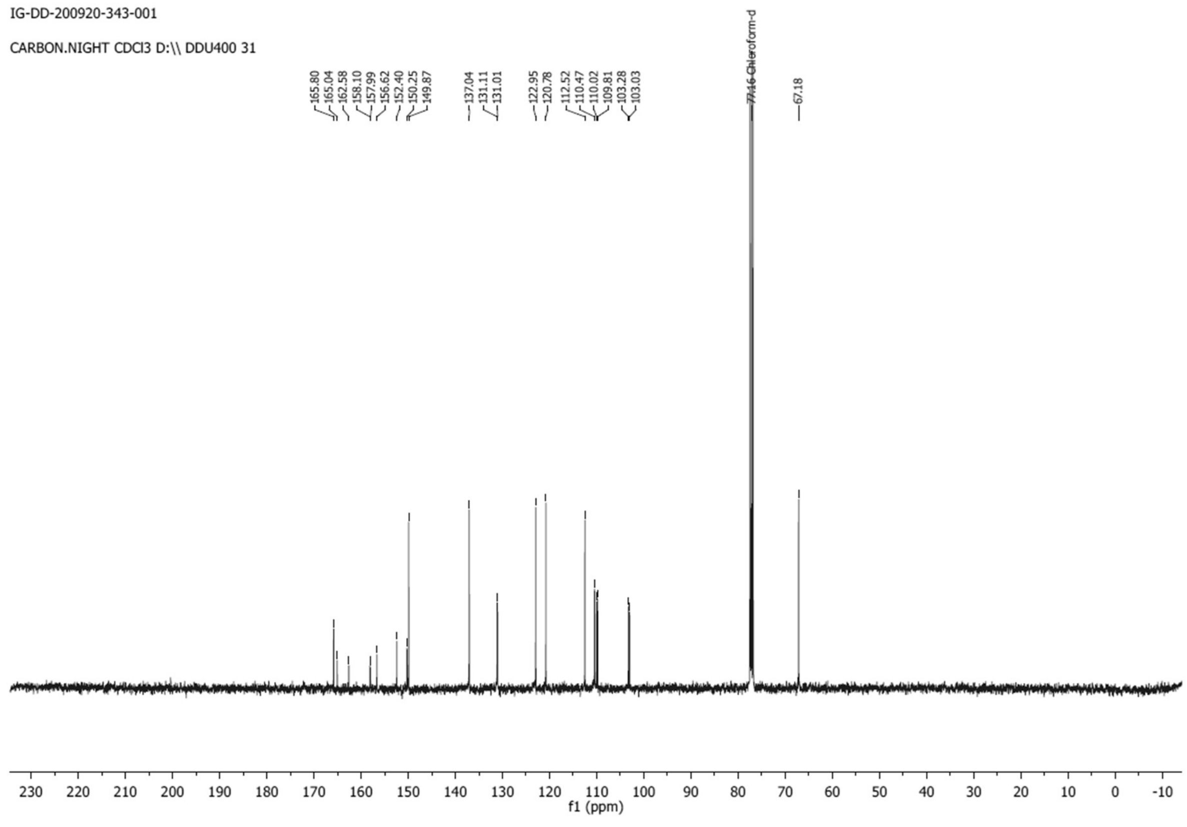

Figure S11: ^{13}C NMR (101 MHz, $CDCl_3$) of **3a**

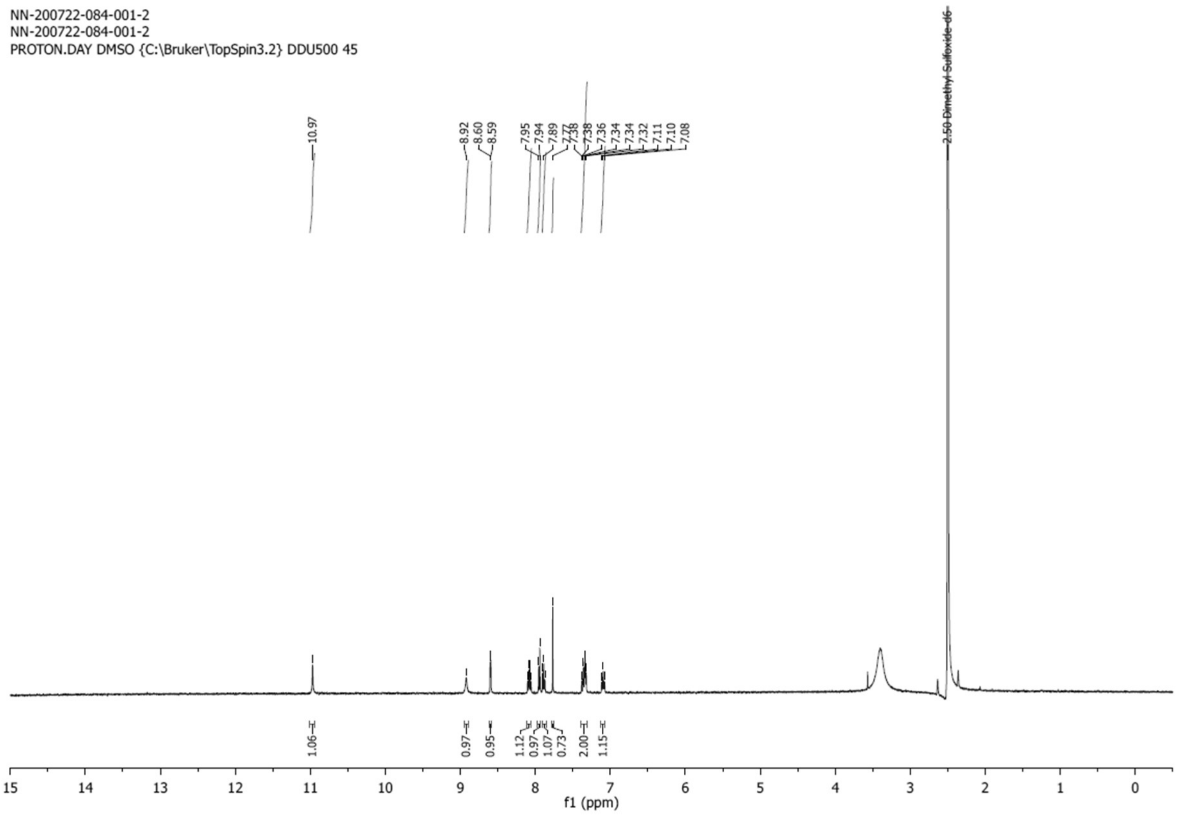

Figure S12: ^{1}H NMR (500 MHz, DMSO-d^6) of **3b**

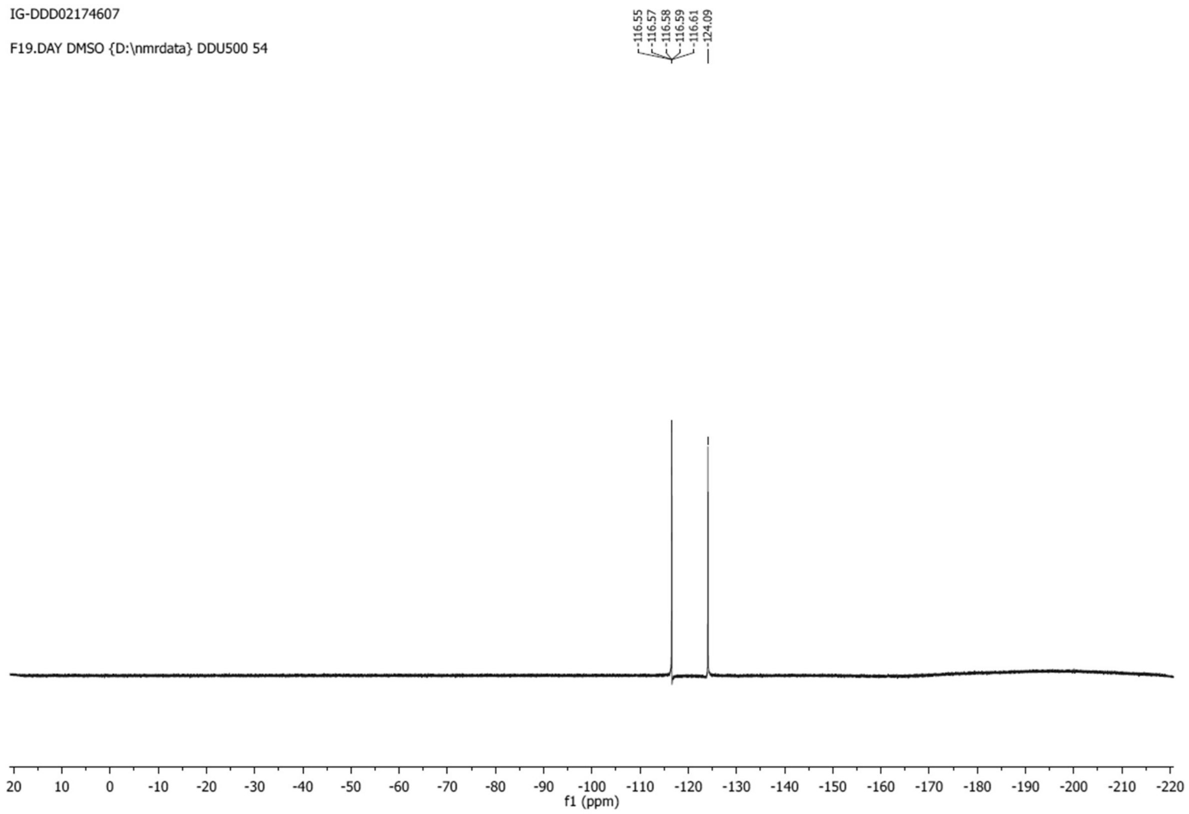

Figure S13: ^{19}F NMR (471 MHz, DMSO-d^6) of **3b**

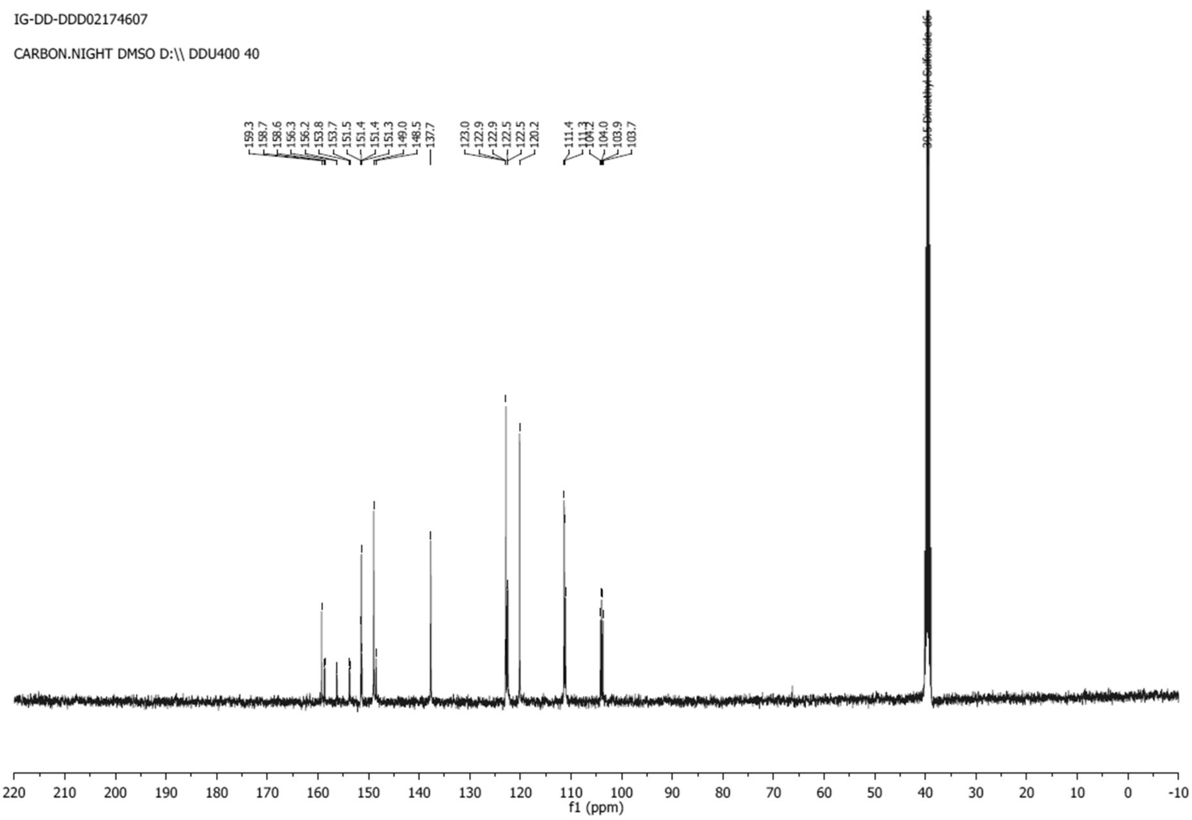

Figure S14: ^{13}C NMR (101 MHz, DMSO-d^6) of **3b**

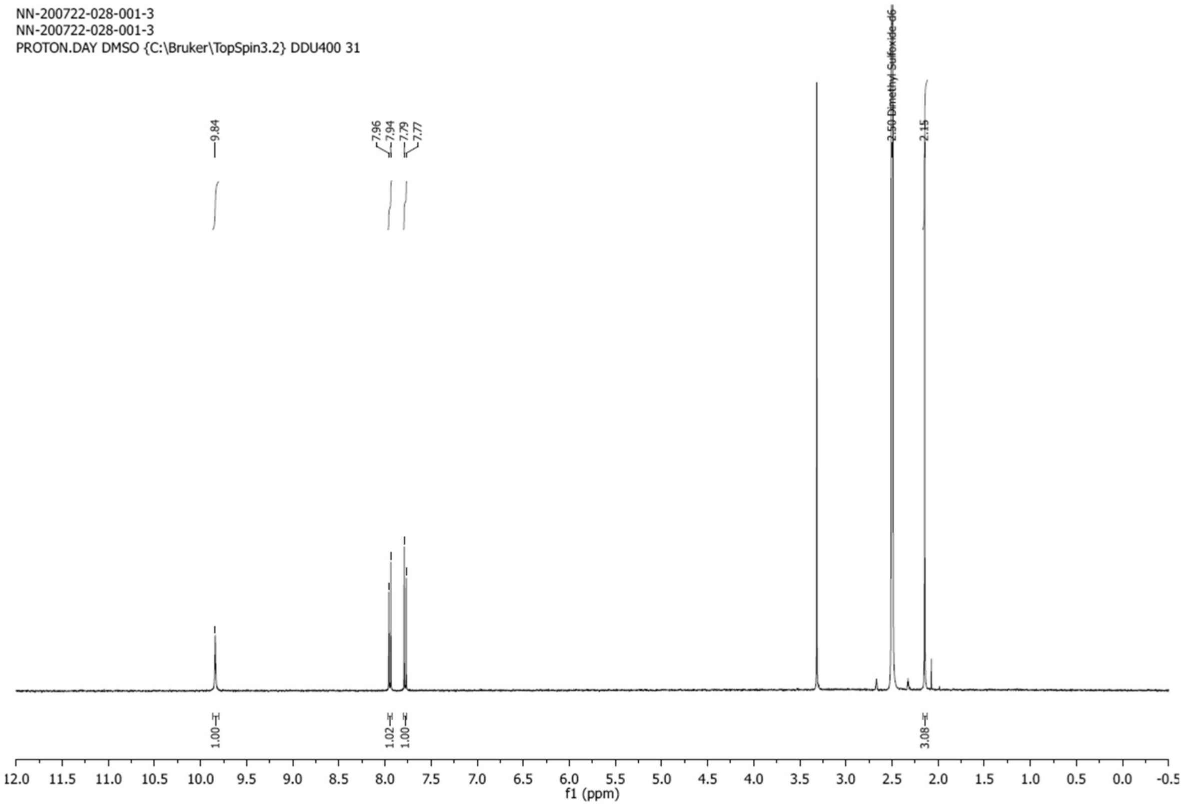

Figure S15: ^{1}H NMR (400 MHz, DMSO-d^6) of **4a**

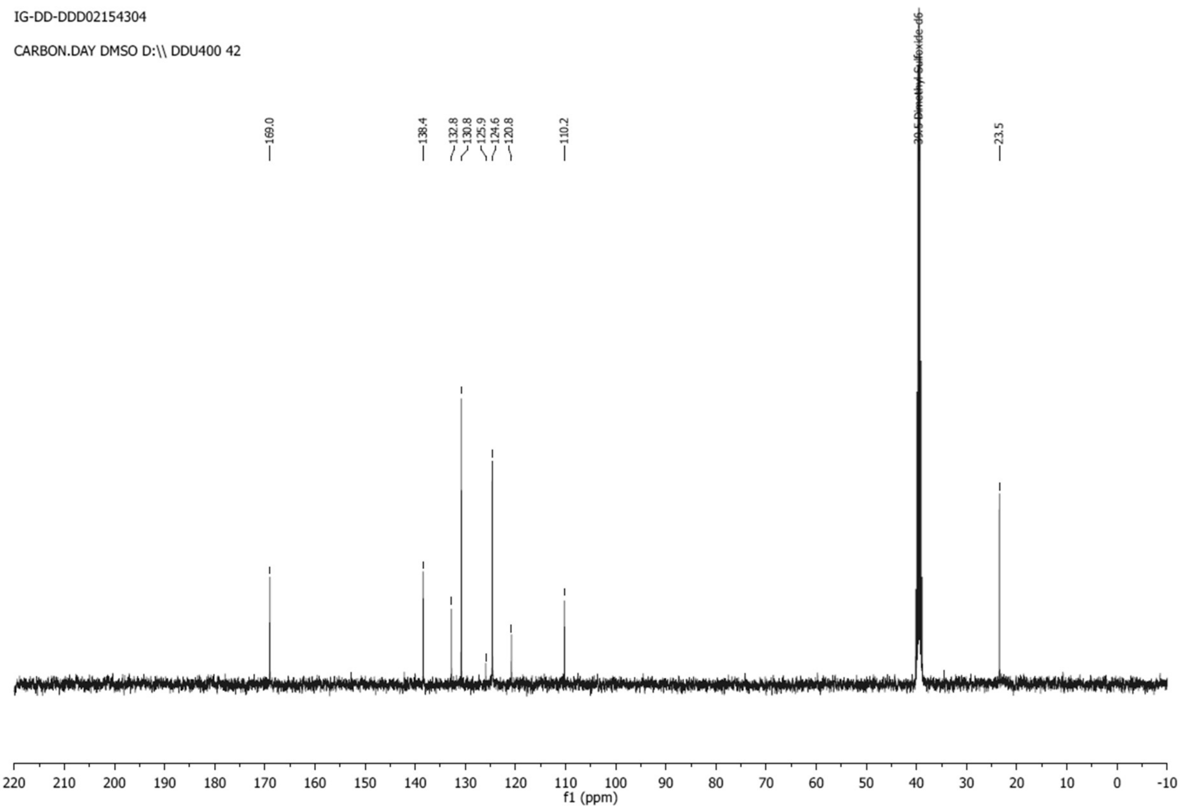

Figure S16: ^{13}C NMR (400 MHz, DMSO-d^{6}) of **4a**

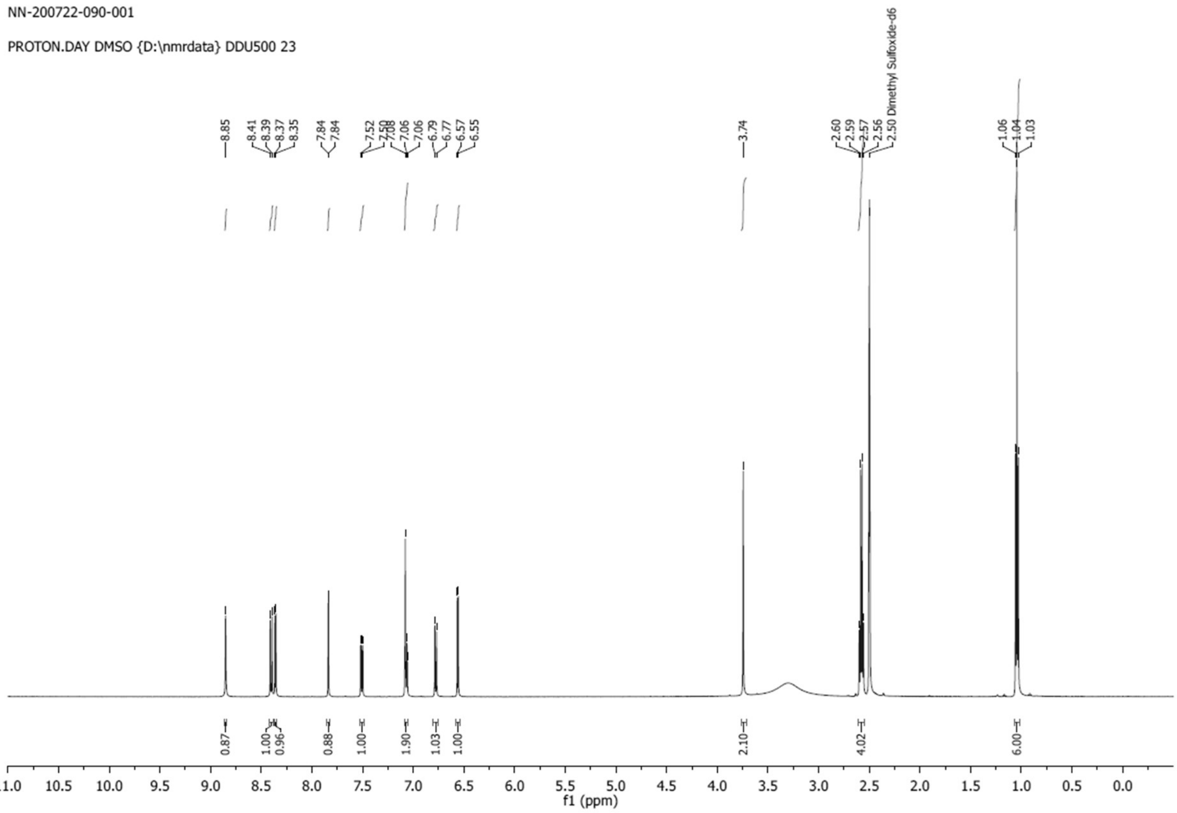

Figure S17: ^{1}H NMR (500 MHz, DMSO-d^{6}) of **5a**

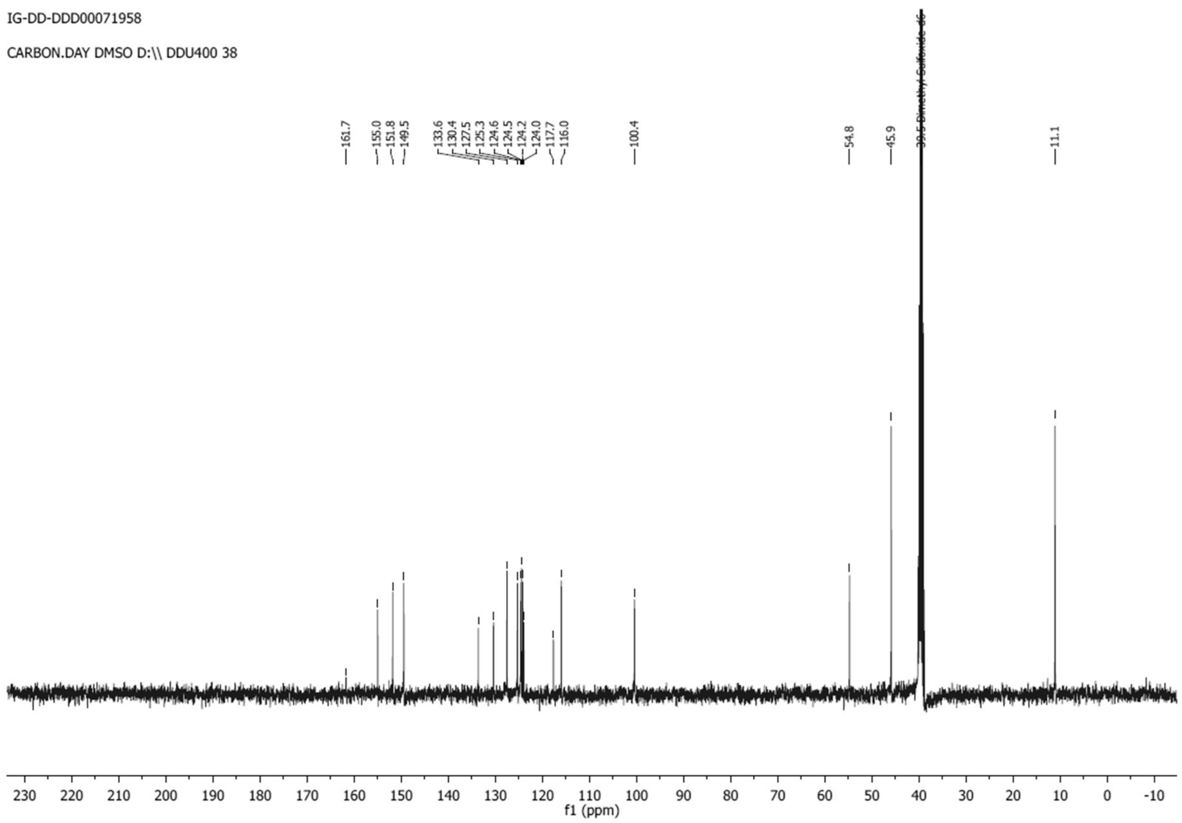

Figure S18: ^{13}C NMR (100 MHz, DMSO-d^6) of **5a**

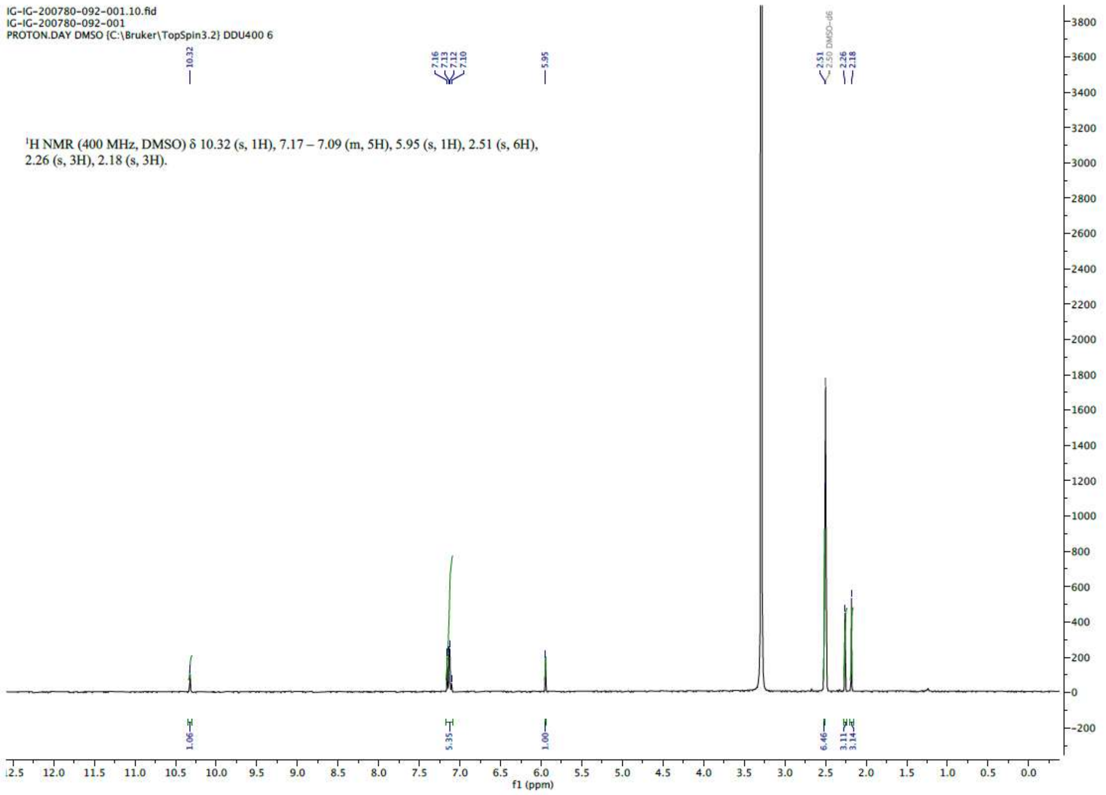

Figure S19: ^{1}H NMR (400 MHz, DMSO-d^6) of **6b**

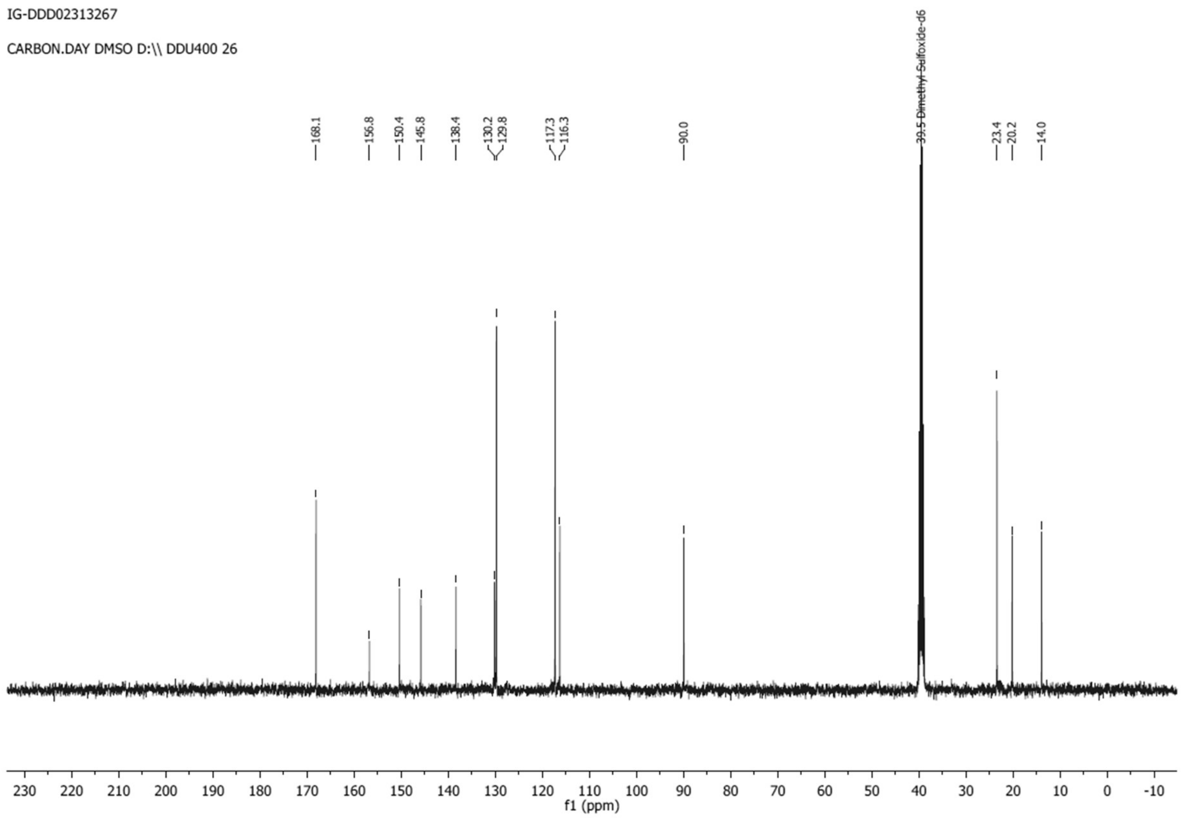

Figure S20: ^{13}C NMR (100 MHz, DMSO-d^{6}) of **6b**

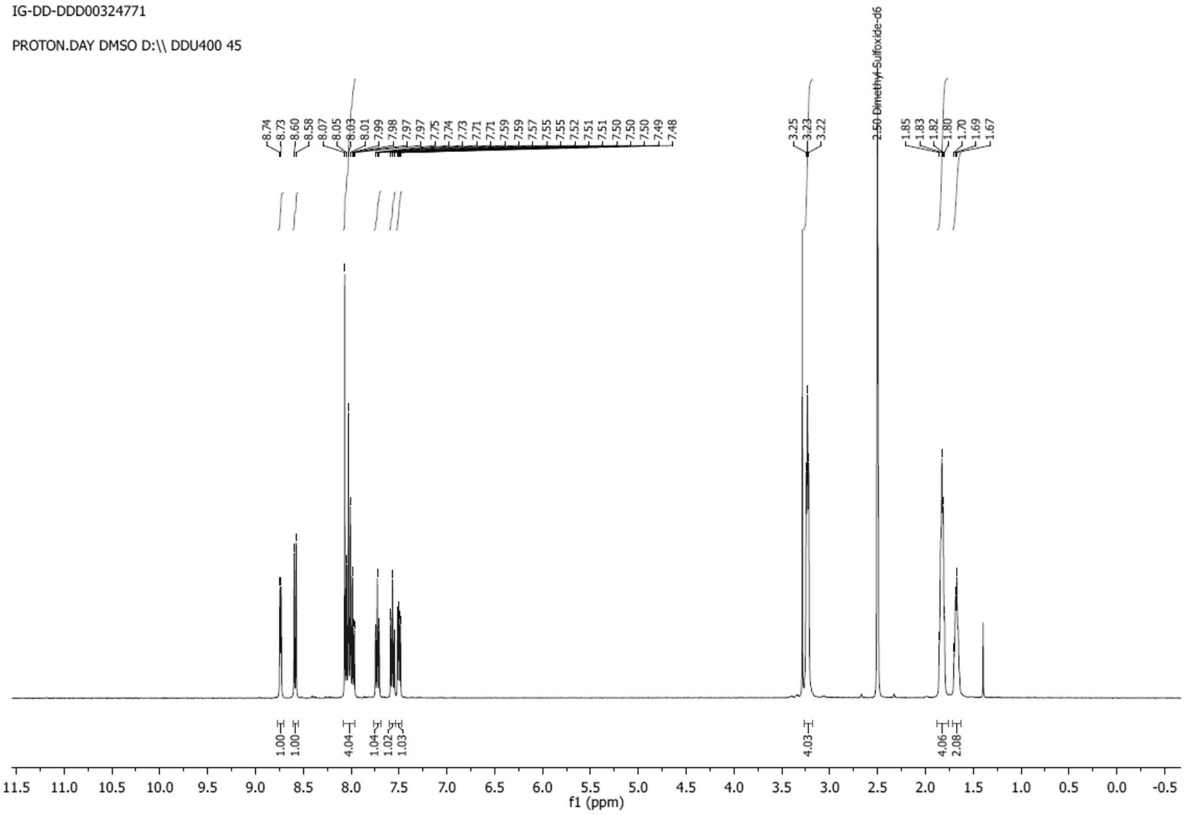

Figure S21: ^{1}H NMR (500 MHz, $CDCl_3$) of **7a**

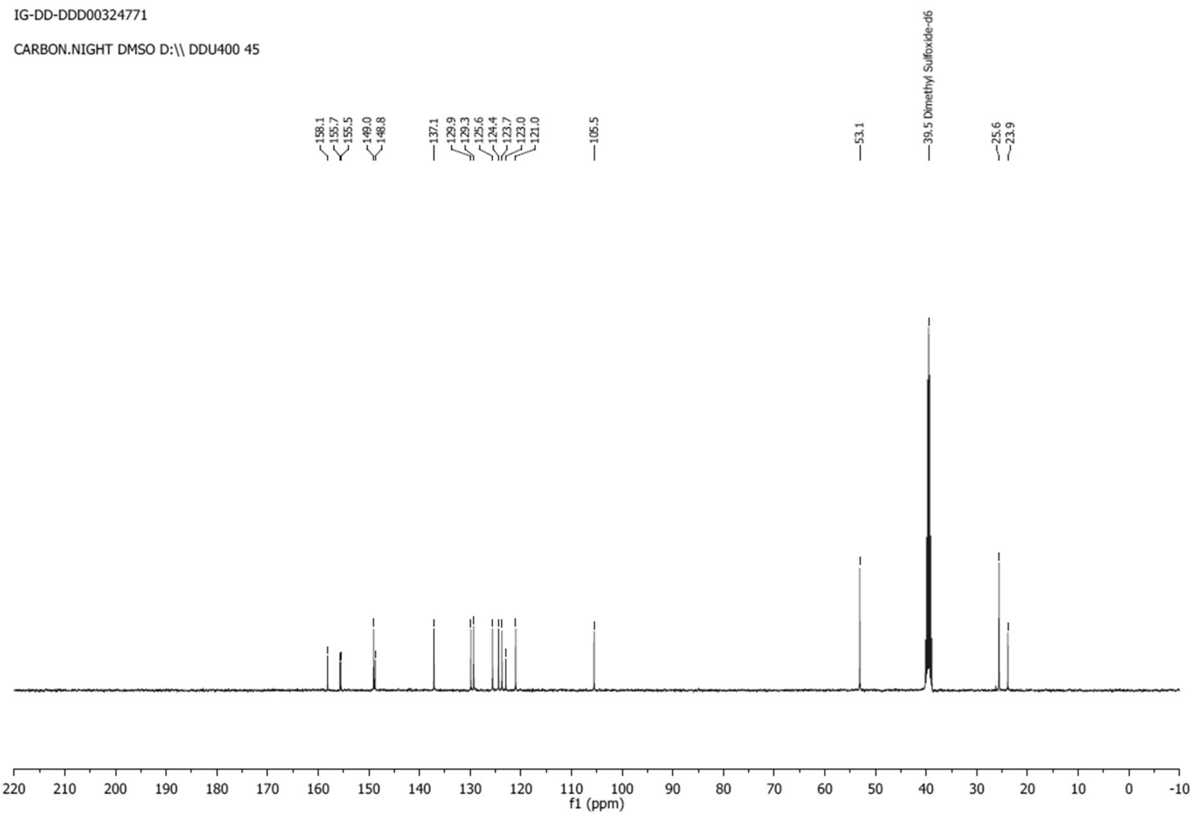

Figure S22: ^{13}C NMR (125 MHz, $CDCl_3$) of **7a**

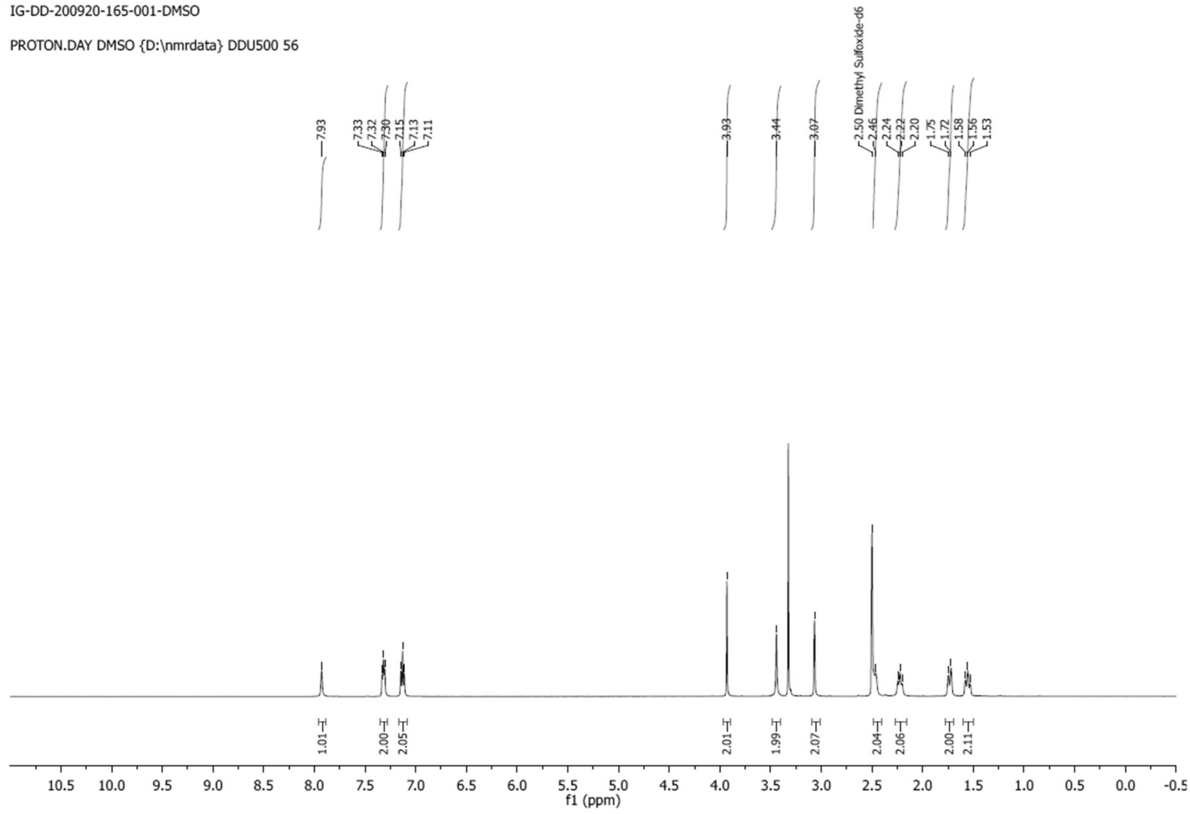

Figure S23: ^{1}H NMR (500 MHz, DMSO-d^6) of **8a**

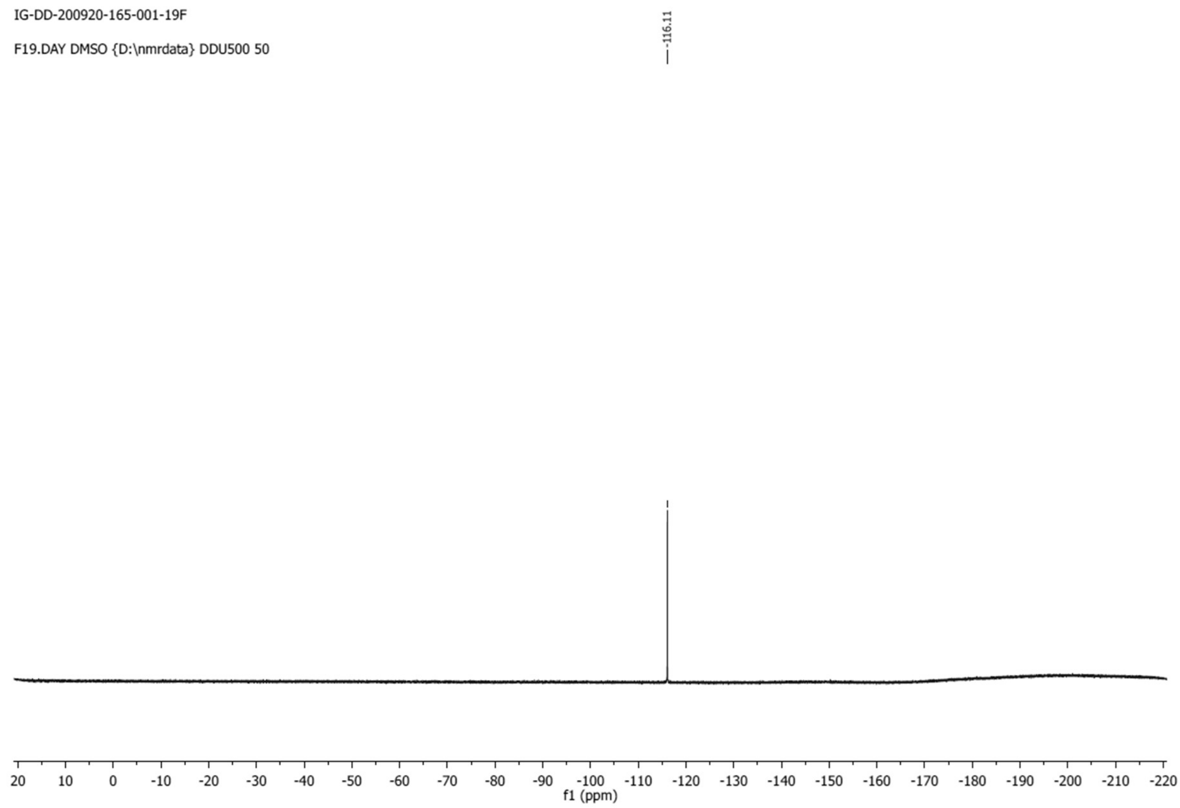

Figure S24: ^{19}F NMR (471 MHz, DMSO-d^6) of **8a**

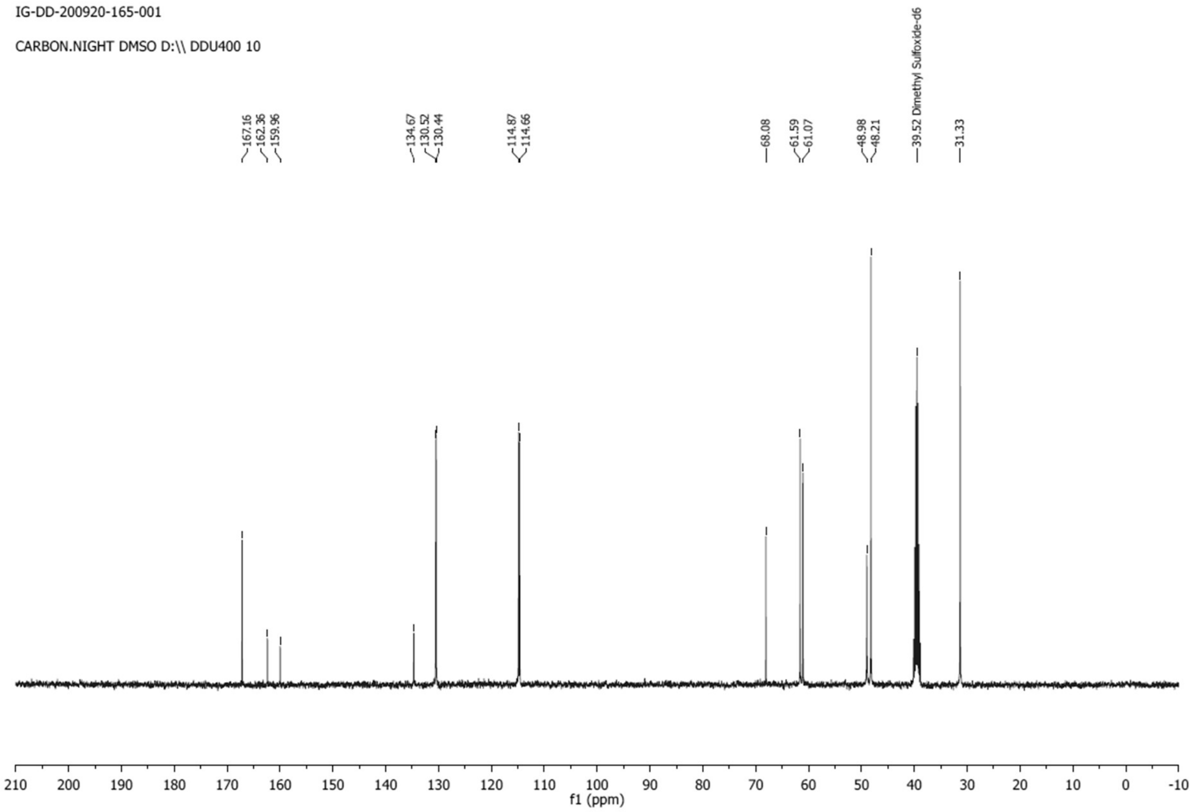

Figure S25: ^{13}C NMR (100 MHz, DMSO-d^6) of **8a**

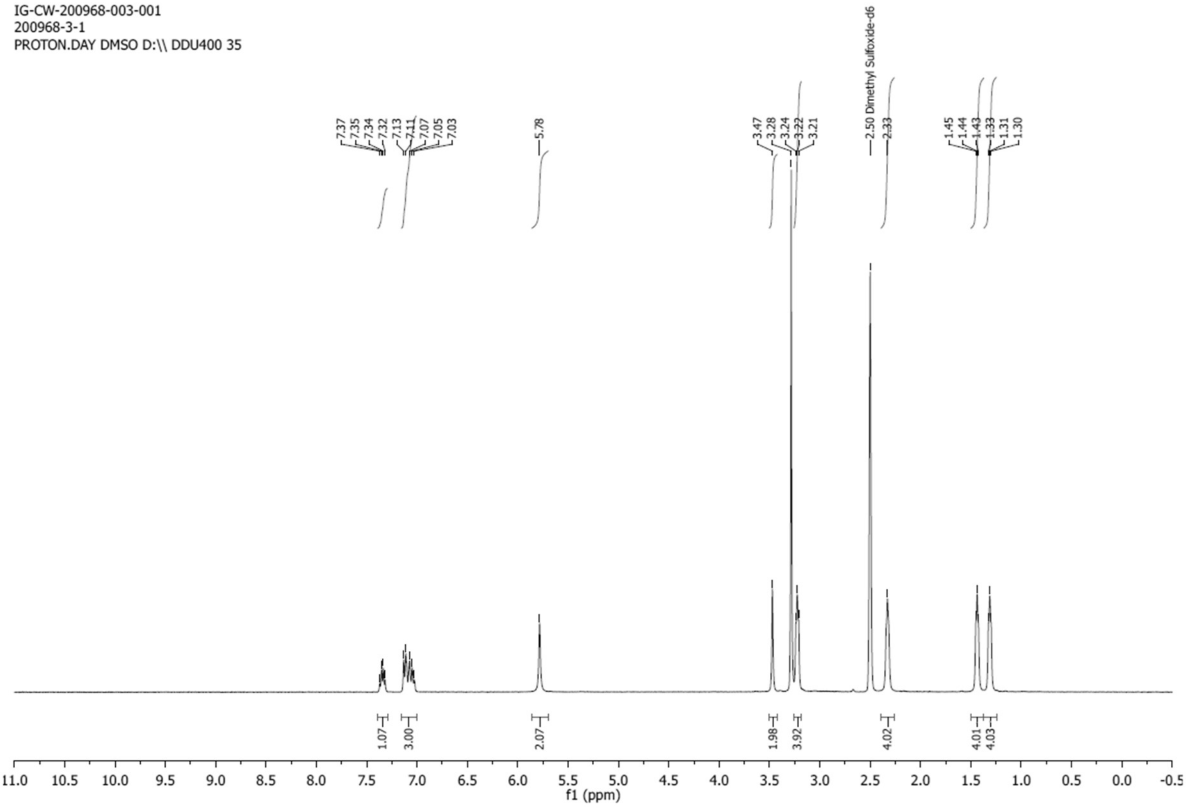

Figure S26: ^{1}H NMR (400 MHz, DMSO-d^{6}) of **9a**

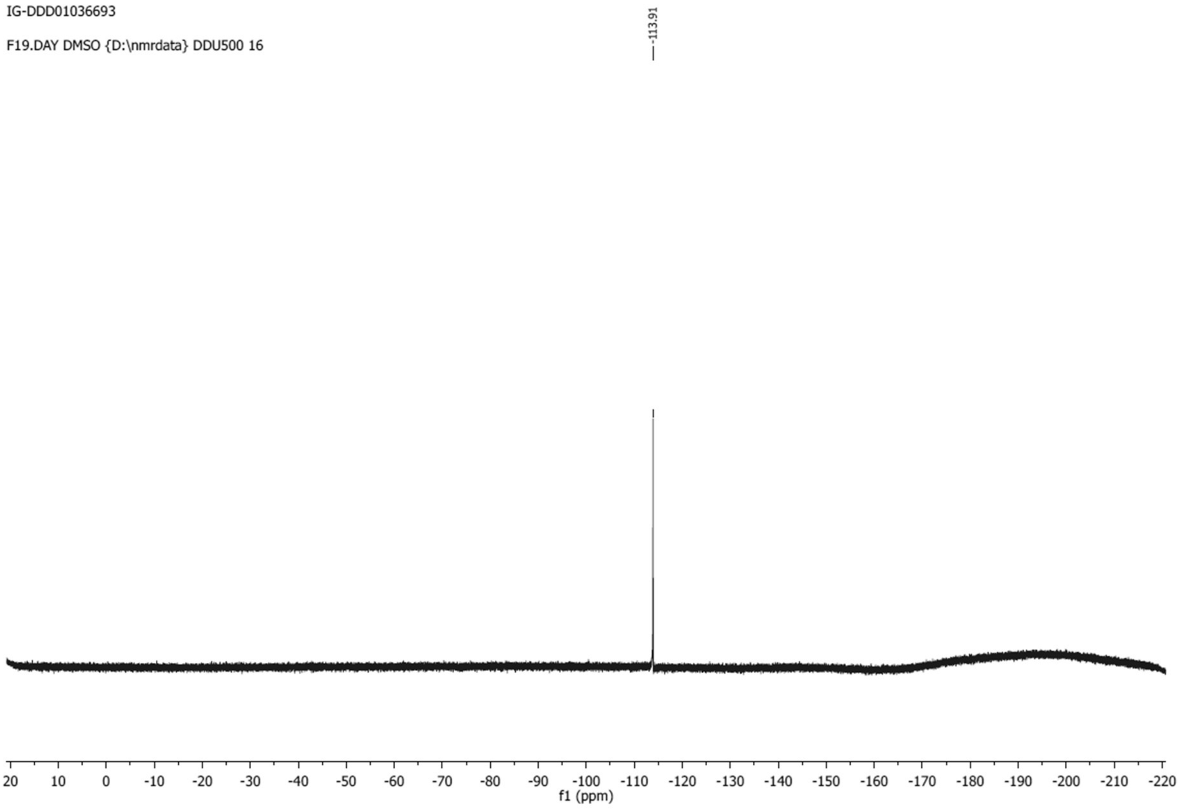

Figure S27: ^{19}F NMR (471 MHz, DMSO-d^{6}) of **9a**

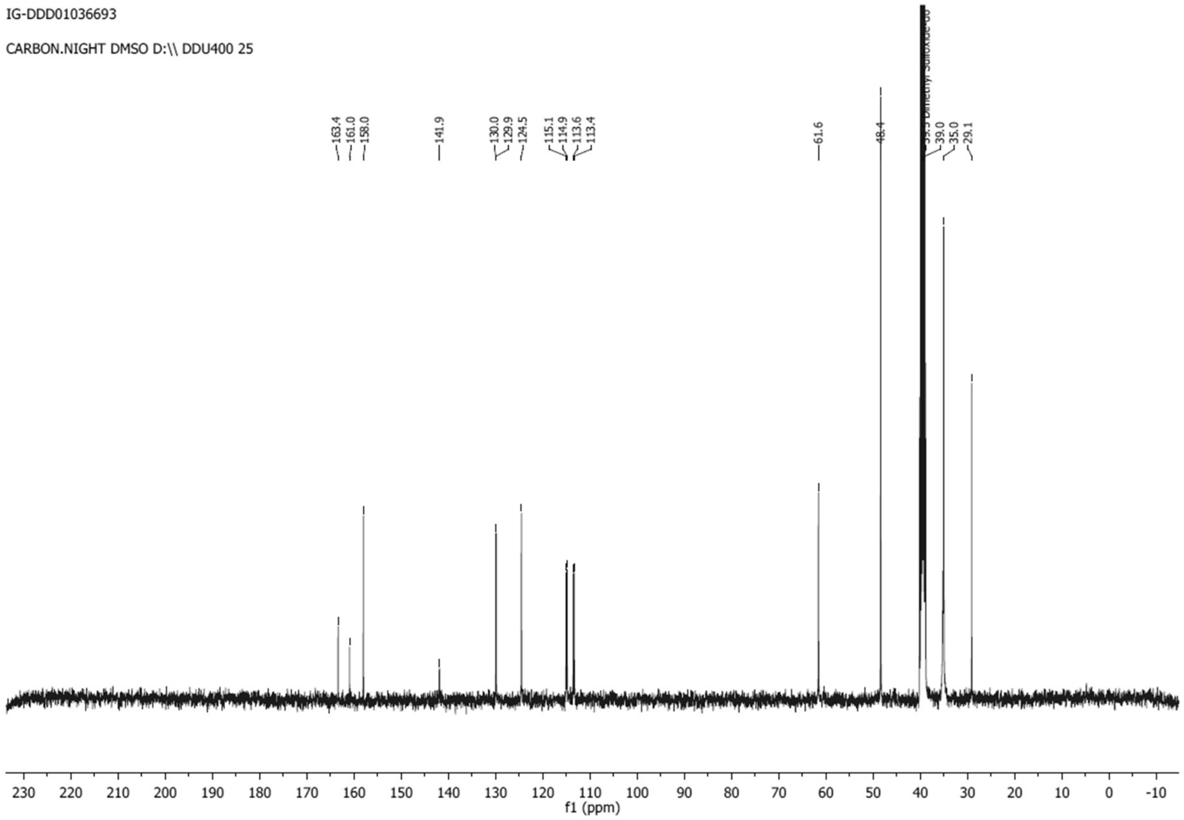

Figure S28: ^{13}C NMR (100 MHz, DMSO-d^6) of **9a**

NMR data of intermediates

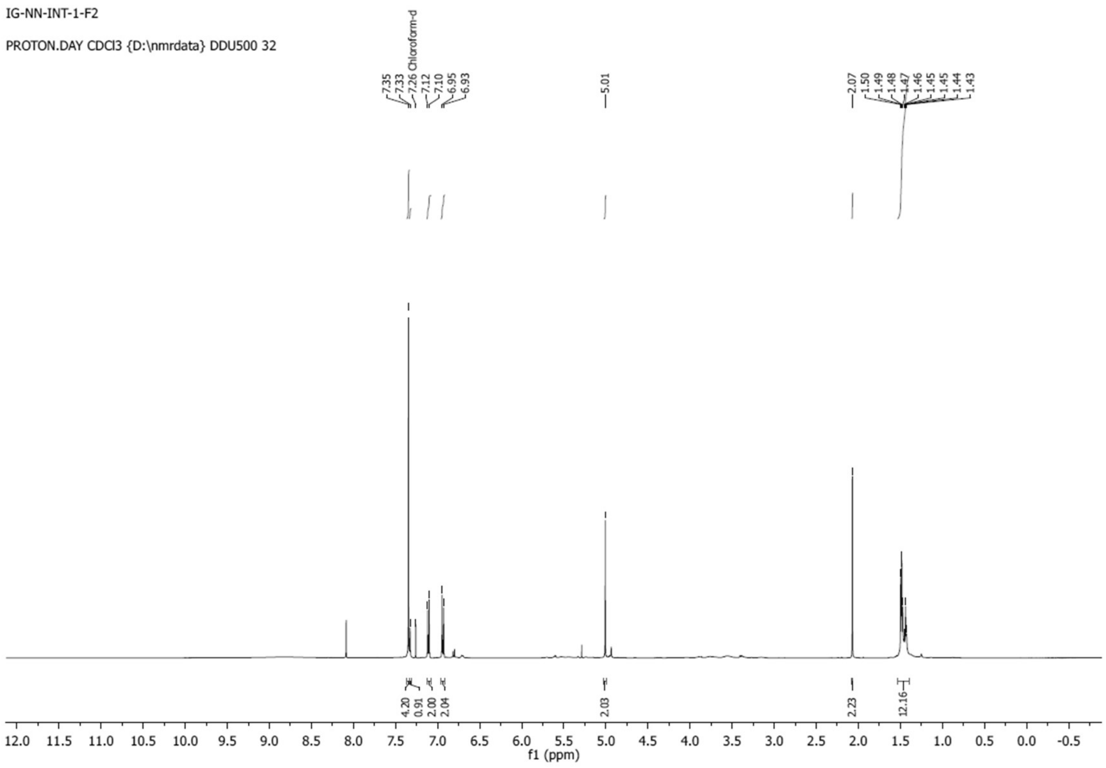

Figure S29: ^{1}H NMR (500 MHz, $CDCl_3$) of **1o**

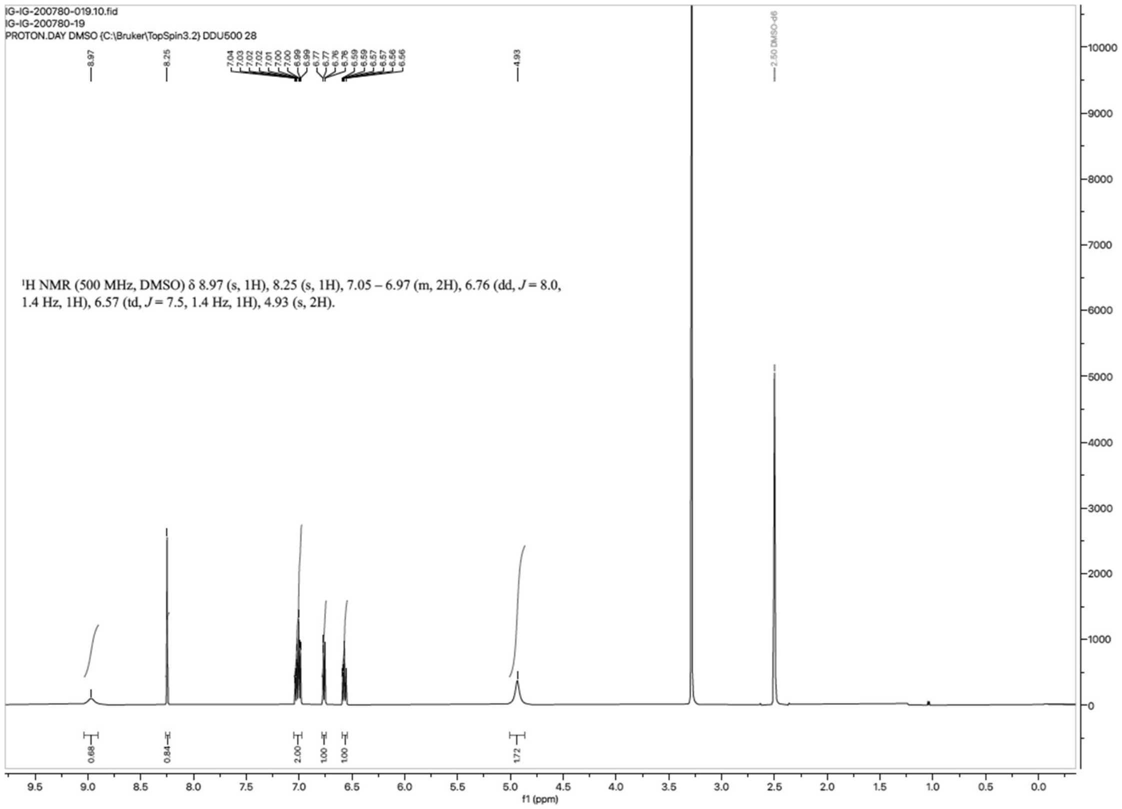

Figure S30: ^{1}H NMR (500 MHz, DMSO-d^6) of **2g**

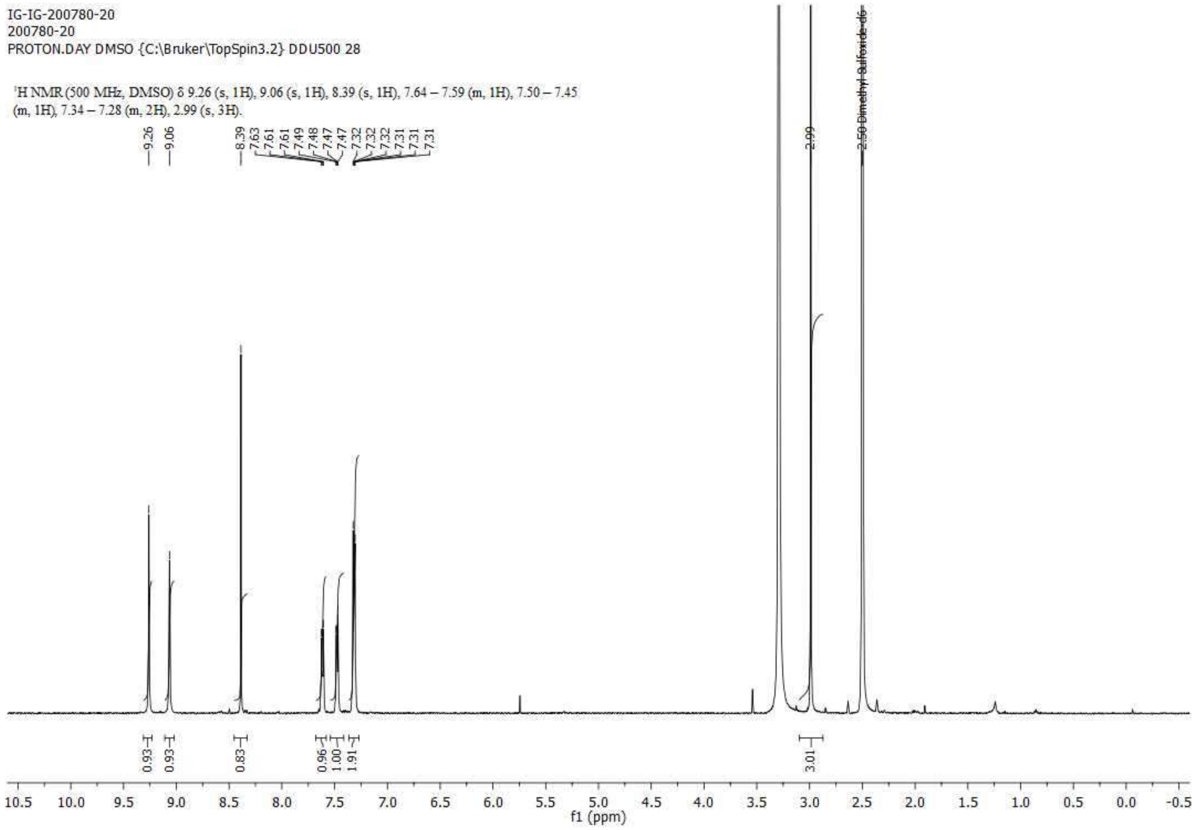

Figure S31: ^{1}H NMR (500 MHz, DMSO-d^6) of **2h**

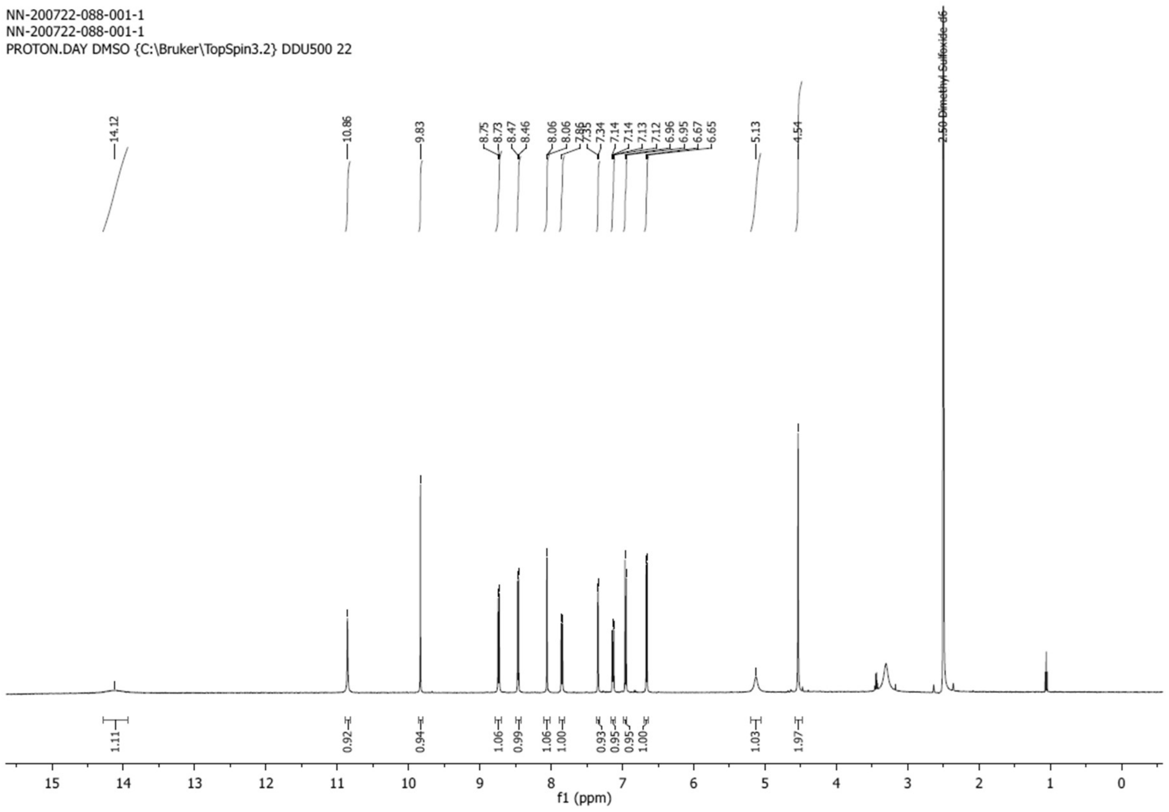

Figure S32: ^{1}H NMR (500 MHz, DMSO-d^6) of **5l**

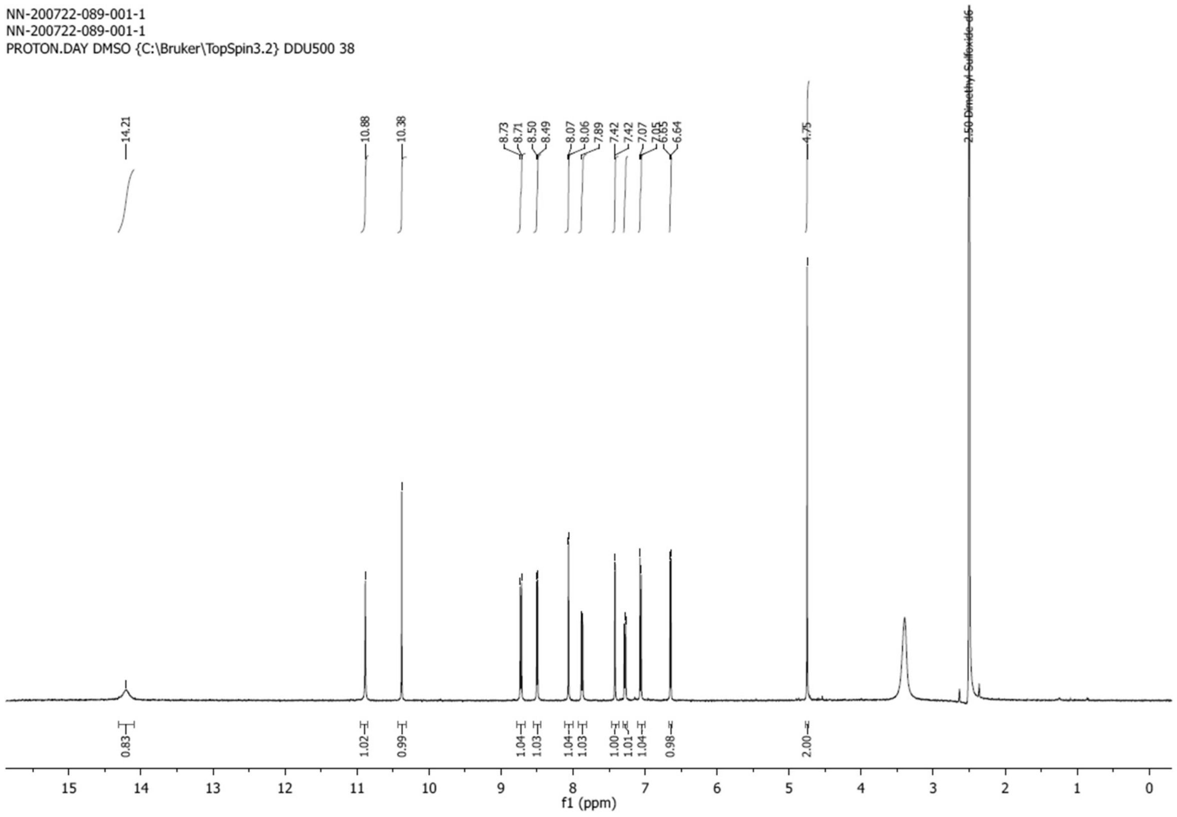

Figure S33: ^{1}H NMR (500 MHz, DMSO-d^6) **of 5m**
